# Gene expression analysis of Atlantic salmon gills reveals mucin 5 and interleukin 4/13 as key molecules during amoebic gill disease

**DOI:** 10.1038/s41598-018-32019-8

**Published:** 2018-09-12

**Authors:** Mar Marcos-López, Josep A. Calduch-Giner, Luca Mirimin, Eugene MacCarthy, Hamish D. Rodger, Ian O’Connor, Ariadna Sitjà-Bobadilla, Jaume Pérez-Sánchez, M. Carla Piazzon

**Affiliations:** 1 Marine and Freshwater Research Centre, Galway-Mayo Institute of Technology, Dublin Road, Galway, Co., Galway, H91 T8NW Ireland; 2 FishVet Group Ireland, Unit 7b Oranmore Business Park, Oranmore, Co, Galway, H91 XP3F Ireland; 3 Nutrigenomics and Fish Growth Endocrinology Group, Instituto de Acuicultura Torre de la Sal (IATS-CSIC), Castellón, 12595 Spain; 4 Fish Pathology Group, Instituto de Acuicultura Torre de la Sal (IATS-CSIC), Castellón, 12595 Spain

## Abstract

Amoebic gill disease (AGD) is one of the main diseases affecting Atlantic salmon (*Salmo salar* L.) mariculture. Hallmarks of AGD are hyperplasia of the lamellar epithelium and increased production of gill mucus. This study investigated the expression of genes involved in mucus secretion, cell cycle regulation, immunity and oxidative stress in gills using a targeted 21-gene PCR array. Gill samples were obtained from experimental and natural *Neoparamoeba perurans* infections, and sampling points included progressive infection stages and post-freshwater treatment. Up-regulation of genes related to mucin secretion and cell proliferation, and down-regulation of pro-inflammatory and pro-apoptotic genes were associated with AGD severity, while partial restoration of the gill homeostasis was detected post-treatment. Mucins and Th2 cytokines accoun ted for most of the variability observed between groups highlighting their key role in AGD. Two mucins (*muc5*, *muc18*) showed differential regulation upon disease. Substantial up-regulation of the secreted *muc5* was detected in clinical AGD, and the membrane bound *muc18* showed an opposite pattern. Th2 cytokines, *il4/13a* and *il4/13b2*, were significantly up-regulated from 2 days post-infection onwards, and changes were lesion-specific. Despite the differences between experimental and natural infections, both yielded comparable results that underline the importance of the studied genes in the respiratory organs of fish, and during AGD progression.

## Introduction

Gill diseases, caused by infectious and non-infectious agents, are one of the main health challenges for marine Atlantic salmon (*Salmo salar* L.) aquaculture worldwide^1–5^. Amoebic gill disease (AGD), caused by the marine parasite *Neoparamoeba perurans*, is present in all salmon producing countries, and its impact has increased drastically in recent years due to the expansion of its geographic distribution and host range^4,6,7^. Fish suffering from AGD display a marked hyperplasia and fusion of the lamellar epithelium, increased number of goblet cells, and increased production of gill mucus. Lesions are visible macroscopically as white raised mucoid patches in the gill filaments^8,9^. At present, no preventive measures exist, and freshwater or hydrogen peroxide baths are the only available treatments. The success of freshwater treatment is attributed to the osmotic shock on amoebae, together with hydration and subsequent reduction of gill mucus viscosity. Decreased mucus viscosity facilitates the removal of amoebae and excess mucus with the water flow^10,11^. Epithelial proliferation and increased mucus secretion are common hallmarks of many gill diseases, but the molecular mechanisms underlying these pathological changes are not well understood. Characterization of the proliferative host response is important since it ultimately leads to respiratory distress in affected fish, and can result in mortalities if left untreated.

Mucus covering epithelial surfaces is composed of mucins and a complex mixture of proteins, enzymes, ions and lipids^12^. Mucins, the most abundant components of mucus, are high molecular weight glycoproteins secreted by goblet cells and described to play important roles in innate immunity, accommodation of the commensal flora, and limitation of pathogens^13^. Mucins are classified into secreted (mostly gel-forming) or membrane-bound, and to date more than 20 mucin genes have been identified in higher vertebrates. In humans, mucins have been extensively studied and are used as biomarkers for several diseases, including cancer and airway disorders^14,15^. The wealth of data available from mammals contrasts with the limited data existing for fish mucins. The large size and repetitive sequence of mucins peptidic chains complicate their characterization. Nonetheless, the recent availability of extensive sequence databases can be a useful resource to identify and study these molecules, particularly in lower vertebrates where information is scarce. Mucin genes in fish have been described for a small number of teleost species including zebrafish (*Danio rerio*)^16,17^, gilthead sea bream (*Sparus aurata*)^18^, common carp (*Cyprinus carpio*)^19^, and sea trout (*Salmo trutta*)^20^. In Atlantic salmon, many mucin sequences can be found as predicted sequences in the databases based on homology with other species, but only recently, gene expression analyses have been carried out to detect expression of mucins in mucosal tissues of this species^21^. Prior to this, there was only one report identifying mucin gene expression in skin of Atlantic salmon^22^.

Previous studies on AGD gene expression have largely focused on immune genes^23–27^ or microarray-based transcriptome profiling^28–30^. Based on this information, the present study was designed as a targeted approach pinpointing genes of interest belonging to pathways involved in mucus secretion, cell proliferation and apoptosis, and oxidative/cellular stress during an experimentally controlled AGD infection and a natural outbreak. Immune markers known to be differentially expressed in AGD^23^ (*tnfα3*, *il4/13a*, *il4/13b2* and *tgfβ1b*), but also related to mucin secretion or cell hyperplasia, were as well included. The rationale for the selection of genes was also supported by previous proteomic^31^ studies, and known gene functions in other animal species; such as stimulation of mucin secretion (MUC5AC/MUC5B) by Th2 interleukins^32,33^ and epidermal growth factor receptor (EGFR)^34,35^ in mammals, or the important role of mitogen activated protein kinase (MAPK) pathways and Krüppel-like factors (KLFs) in cell cycle regulation^36,37^. Sequential gill samples comprising progressive infection and disease stages were obtained from an *in vivo N. perurans* bath challenge and from a natural infection in a commercial Atlantic salmon farm. For the field trial, samples were also collected post-freshwater treatment and gill samples were further divided into lesion and non-lesion areas. The results obtained contribute to the ongoing understanding of AGD host response and pathogenesis and identify key molecules involved in gill health.

## Results

### *In vivo* challenge

**Figure 1:**
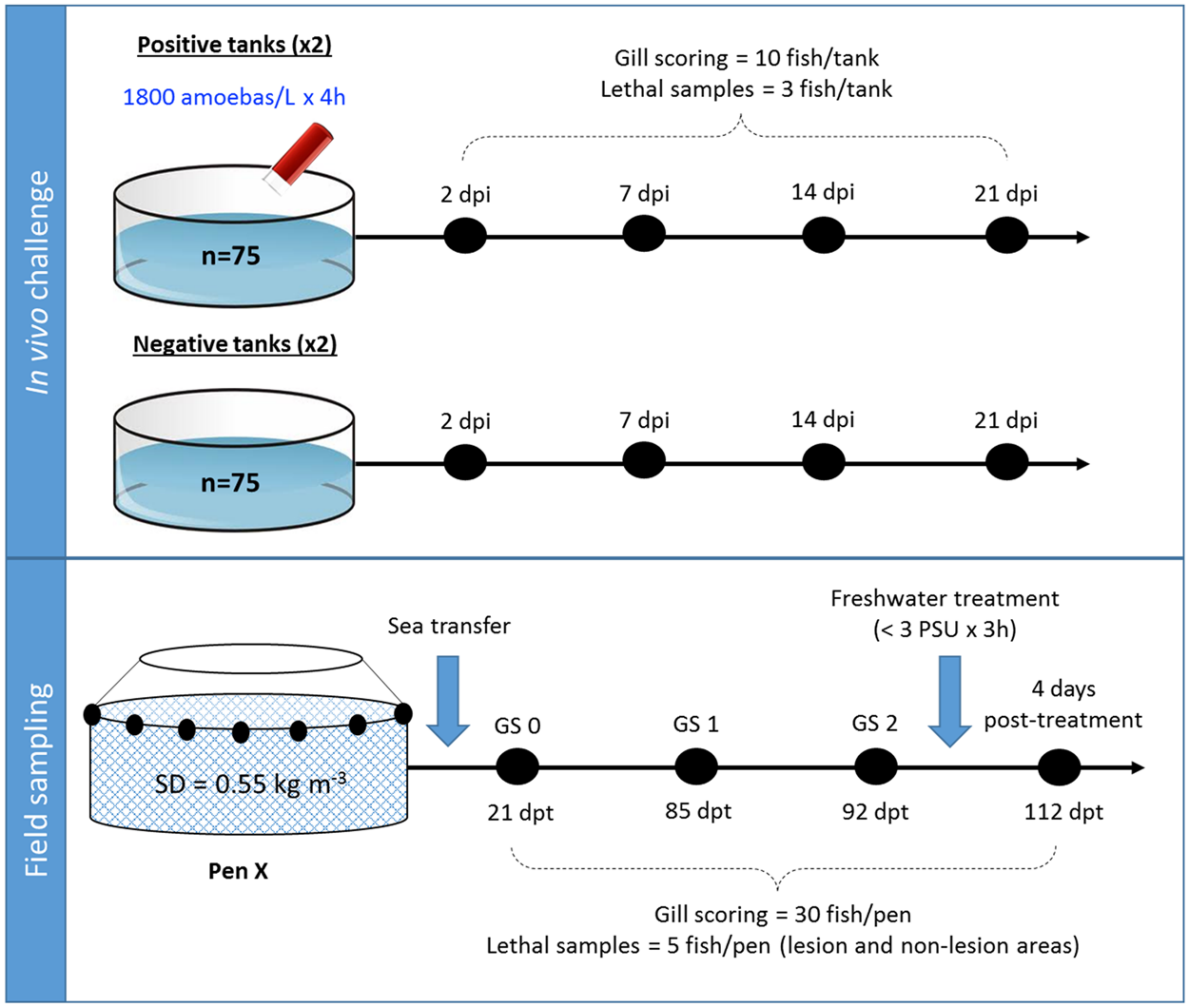
Schematic representation of the two different trials (*in vivo* challenge with *Neoparamoeba perurans* and field sampling) used in this study. SD = initial stocking density. Black dots represent the sequential sampling points (dpi = days post-infection; dpt = days post-transfer). Blue arrows in the field sampling section show initial sea transfer and posterior freshwater treatment. Details on type of lethal samples taken are described in the material and methods section.

**Figure 2:**
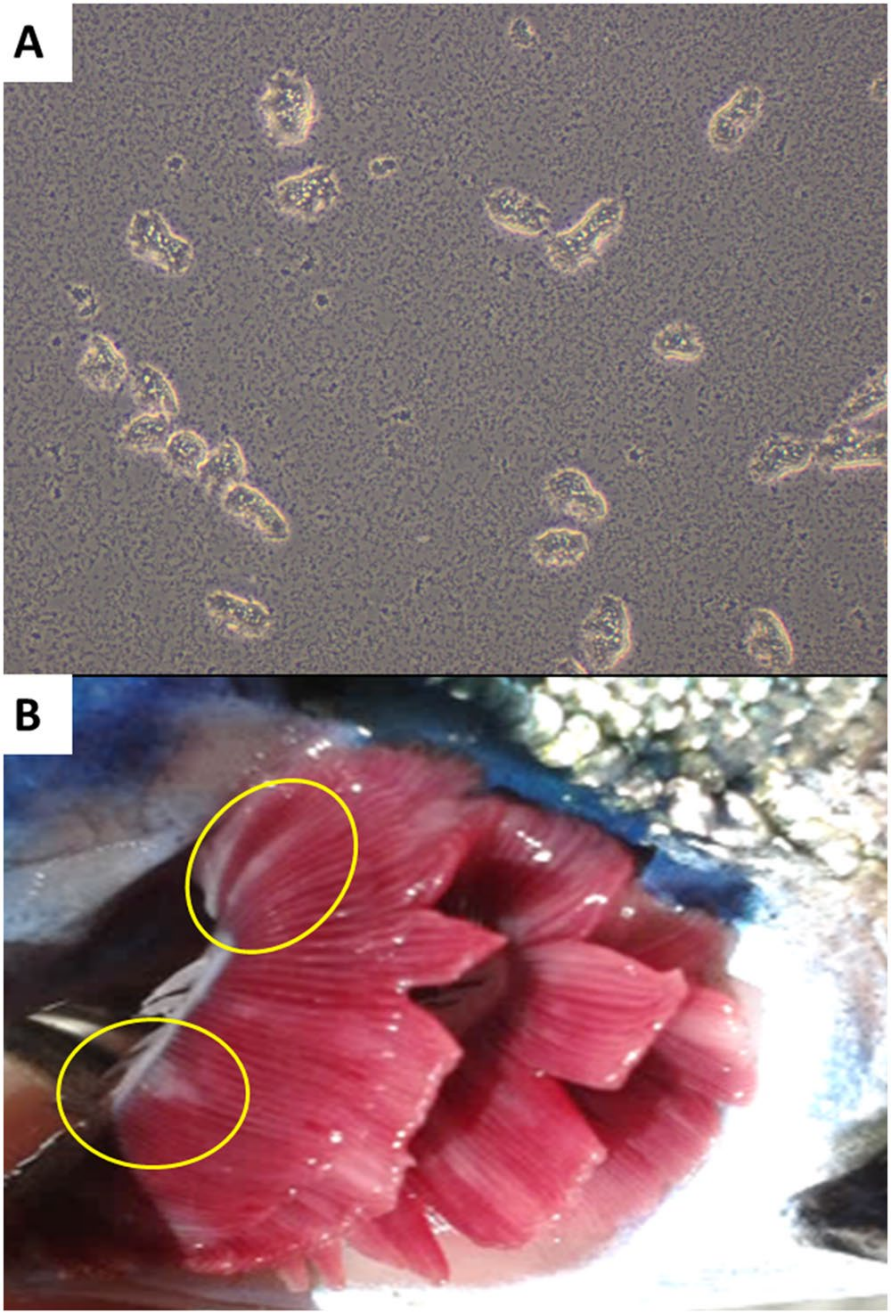
(**A**) Cultured *Neoparamoeba perurans* in marine malt yeast agar plates covered with filtered and sterile seawater (x400). (**B**) Macroscopic AGD lesions in experimentally infected fish sampled at 21 dpi. Note mucoid patches at base of filaments (yellow circles).

**Figure 3:**
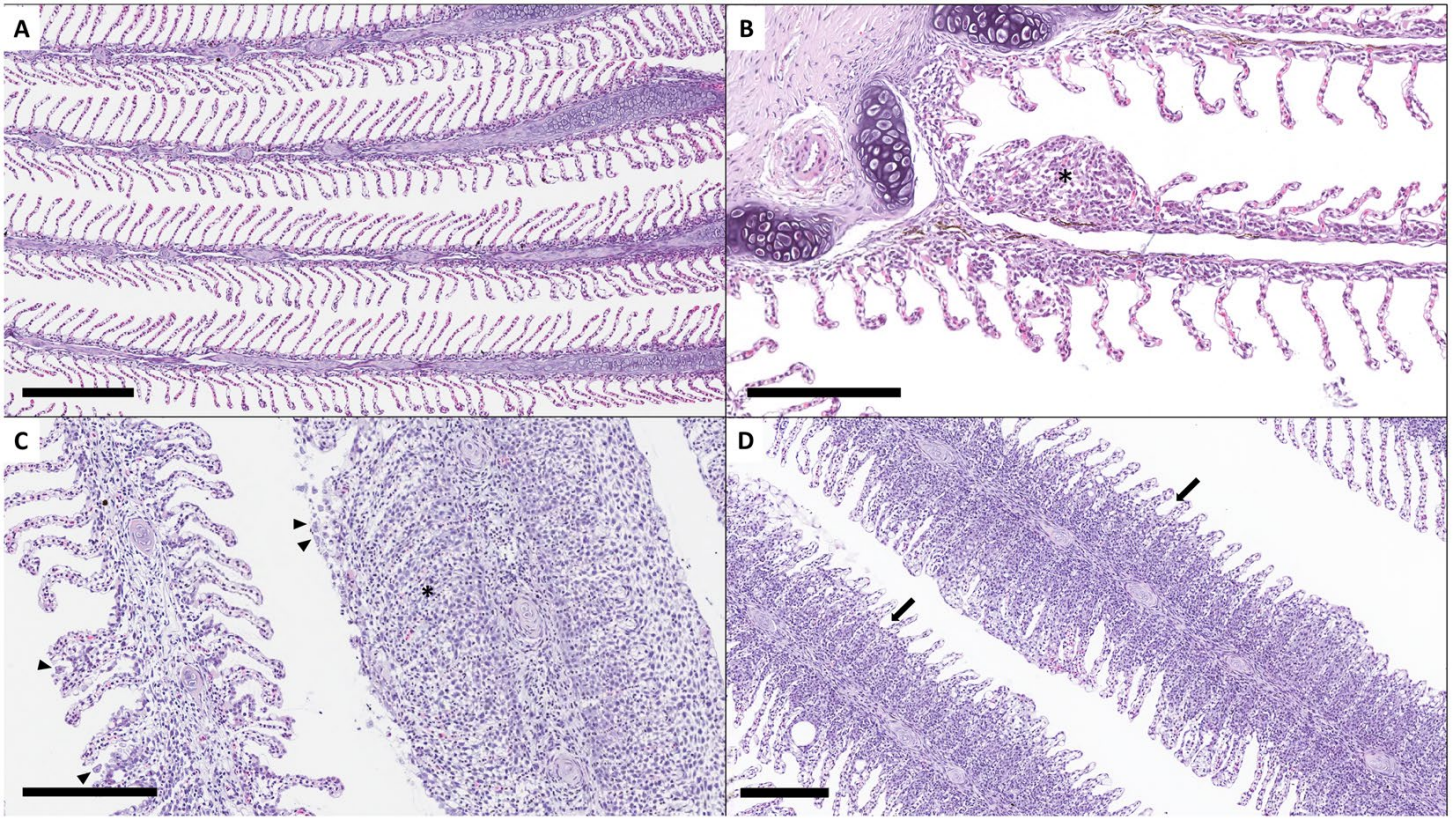
Histopathology assessment of AGD infection (H&E staining). (**A**) Sample from negative control fish at 2 dpi showing non-infected gills with normal lamellar structure. Scale bar = 300 μm. (**B**) Sample from infected fish at 2 dpi showing infection signs such as mild focal hyperplasia and fusion of lamellar epithelium (*). Scale bar = 200 μm. (**C**) Sample from GS 2 fish (field sampling) showing moderate multifocal hyperplasia and fusion of lamellar epithelium (*) with associated presence of amoeboid organisms (arrow heads). Scale bar = 200 μm. (**D**) Histology sample taken 4 days after freshwater treatment with less prominent hyperplastic foci. Note regression of inter-lamellar cell mass (arrows). Scale bar = 200 μm.

An experimental *in vivo* challenge was performed to assess the gill response upon infection under controlled conditions. The details of the *in vivo* challenge are summarized in Fig. 1. Cultured *N. perurans* collected from naturally infected fish (Fig. 2A) were used to infect by bath challenge naïve Atlantic salmon smolts. Gill scoring (Fig. 2B) and lethal samples were taken after 2, 7, 14 and 21 days post-infection (dpi). Macroscopically, challenged fish did not show gross AGD lesions at 2 and 7 dpi (overall gill score (GS) of 0), and showed average gill scores (SEM) of 1.9 (0.18) and 3.3 (0.21) at 14 and 21 dpi, respectively (Fig. 2B). Histopathological assessment of infected samples revealed mild foci of epithelial hyperplasia at 2 dpi, and microscopic lesions consistent with AGD (i.e. multifocal hyperplasia and fusion of the lamellar epithelium and presence of amoebae in close association with lesion areas) from 7 dpi onwards (Fig. 3). *N. perurans* was detected by real-time PCR in 50% of the fish sampled at 2 dpi, and in 100% of the fish sampled at 7, 14 and 21 dpi. Samples from non-infected tanks subjected to gill scores, real-time PCR and histopathology analysis, remained negative for the duration of the experiment.

### Field sampling

To determine the response upon natural infection, field samples were obtained from a commercial farm suffering an AGD outbreak. Fish were monitored and sampled from sea transfer to the development of disease signs and subsequent freshwater treatment (Fig. 1). No macro- or microscopic AGD lesions were noted in any of the fish sampled at 21 days post-transfer (dpt) (GS 0), and all fish sampled at GS 0 tested negative for *N. perurans* by PCR. At 85 dpt (GS 1) and 92 dpt (GS 2), all fish obtained for analysis displayed gross AGD lesions consistent with stage 1 or 2 of disease severity, respectively. The average pen gill score (SEM) at the day of sampling was 0.5 (0.09) for GS 1 and 1.7 (0.11) for GS 2. AGD was confirmed by histopathology (Fig. 3C) and all fish sampled at GS 1 and GS 2 tested positive for *N. perurans* by PCR. At the sampling point carried out four days after freshwater treatment (112 dpt), AGD lesions were still visible macroscopically but these appeared to have less depth, less mucus and were paler in colour. Histopathology demonstrated multifocal proliferation of the lamellar epithelium, but hyperplastic foci were composed of fewer lamellae and regression of the inter-lamellar cell mass was apparent in most lesions (Fig. 3D). No amoebae were observed in the histology sections, but two out of the five fish sampled were positive by PCR. No other significant gill pathology was noted by gross or histopathological examination at any of the sampling points.

### Gene sequence analysis

**Figure 4:**
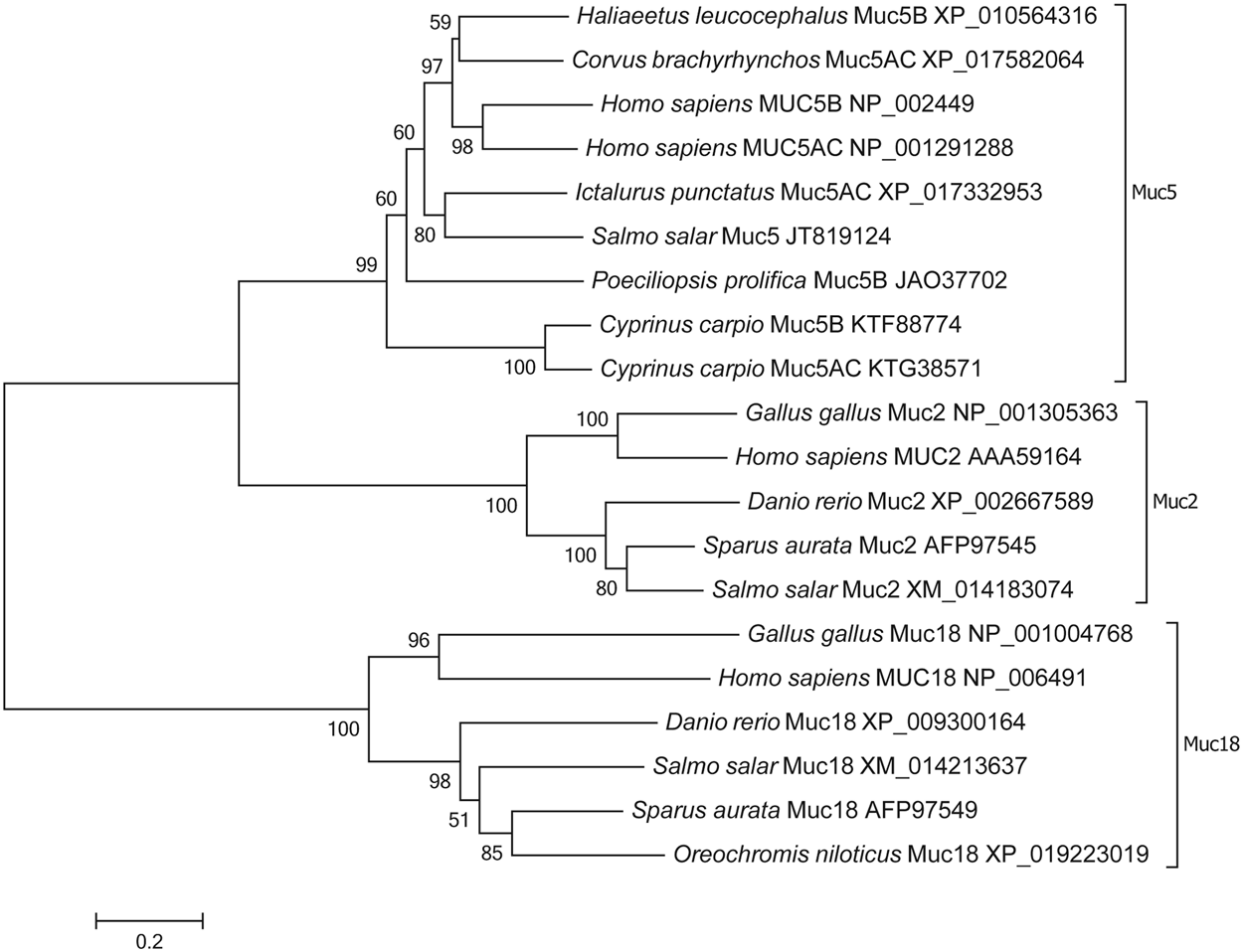
Phylogenetic tree of protein sequences for Muc5, Muc2 and Muc18, including the Atlantic salmon sequences used in the present study. GenBank accession numbers are provided for each sequence.

This study aimed to determine the specific expression dynamics of several targeted genes in Atlantic salmon gills during AGD. To that aim, a 21 gene PCR array was specifically designed to measure the expression of mucins, mitogen-activated protein kinases, Krüppel-like factors, cytokines, markers of oxidative stress, cell proliferation, apoptosis and inflammation, and proteins previously detected to be differentially expressed in AGD in a proteomic study^31^ (Table 1). BLAST searches in the Atlantic salmon genome using mucin sequences described for other fish species resulted in the identification of four significant matches of predicted mucin sequences in Atlantic salmon when using gilthead sea bream mucins. Detected mucins were *muc18* (E-value 1e^−85^) also known as melanoma cell adhesion molecule, *muc2* (E-value 0) annotated in GenBank as intestinal mucin-like, *muc2*-like (E-value 0) and *muc19* (E-value 4e^−75^). Several primers were designed and a preliminary optimization study showed that only *muc18* and *muc2*, together with *muc5* previously detected in Atlantic salmon skin^22^, were expressed in Atlantic salmon gills (data not shown). The phylogenetic tree constructed for Atlantic salmon mucins evidenced the correct classification and grouping of Atlantic salmon mucins with the same type mucins as other teleost and animal species (Fig. 4).

**Table 1:** List of selected genes and forward and reverse primers for real-time qPCR.

Pathway	Gene name (Symbol)	Accession number	Sequence (5′-3′)
Housekeeping	*ß-actin (ß-act)*	AF012125	(F):CCATCCAGGCAGTGTTGT; (R):CGGAGTCCATGACGATACC
Mucins	*mucin 18 (muc18)*	XM_014213637	(F):AAGAGCAGCGAGGTGGTG; (R):TCCGTTGACTTGGCAGATGA
	*mucin 5 (muc5)*	JT819124	(F):CCGTGCTGGGAGACATTATGAAGT; (R):TGCTGGAGAGGGAAAGGGTAAC
	*mucin 2 (muc2)*	XM_014183074	(F):CGACTGCCACAAAGCCATTAGG; (R):GCGTGTTGCTGCGTGTCTT
Immune genes	*tumor necrosis factor α3 (tnfα3)*	NM_001123617	(F):GTGTATGTGGGAGCAGTGTT; (R):GAAGCCTGTTCTCTGTGACT
	*interleukin 4/13a (il4/13a)*	AB574339	(F):GCATCGTTGTGAAGAGCCAAGA; (R):GAAGTCTCCTCAGCTCCACCT
	*interleukin 4/13b2 (il4/13b2)*	HG794525	(F):GTGAAGGAGAACGGTGATGAGGAACAGC; (R):GGCACAGTTGAAGAGGTTTGTCAGGAGAT
	*transforming growth factor β 1b (tgfß1b)*	XM_014129261	(F):GCCATCCGTGGACAGATACT; (R):TCTCCCTCCTGGTCAATCTCT
MAPK pathways	*mitogen-activated protein kinase p38b (p38b)*	EF123660, EF123661	(F):ACGGGTCTGAAGATCGCTGTGAA; (R):TCTGTAGGTTCTCTTGGCGTGGAT
	*extracellular signal–regulated kinase 3 (erk3)*	XM_014176343	(F):CACAGCACTCTGGACACT; (R):ACCTCCTCCTCGTCAGTT
	*c-Jun N-terminal protein kinase 1 (jnk1)*	XM_014158394	(F):GCGGCTGTCCTATCTGCTCTAC; (R):CGGCGTGAAGGTGCTTGATG
Oxidative and cellular stress	*nuclear factor erythroid 2-related factor 2 (nrf2)*	NM_001139807	(F):GAGGGACGAGGATGGGAAG; (R):ATCGGTGGTCTGCTGGAG
	*heat shock 70 kDa protein (hsp70)*	NM_001141684	(F):CCTGCCTACTTCAACGATTCACAGAGACA; (R):CCAGCGATCACTCCAGCGTCCTTA
Cell proliferation	*Prohibitin (phb)*	NM_001140130	(F):GGAGAGGACTACGACGAGAGG; (R):CCACCACGGACTTGAGGAC
	*peptidylprolyl isomerase A (ppia)*	NM_001146606	(F):CTGCTAACACCGACTGGCTGAAC; (R):CTCCACGACCTTGCCGAACAC
	*proliferating cell nuclear antigen (pcna)*	BT056931	(F):GCCGTGACCTGTCTCAGATTG, (R):CCGAGAACTTAACGCCATCCTT
	*epidermal growth factor receptor (egfr)*	XM_014191766	(F):GACACCAAGTTCTACCAGAGCCTAATCAGT; (R):GCGTCCACAGCGTCCTCCAT
Apoptosis	*cellular tumor antigen p53 (p53)*	BT058777	(F):CATCATCACCCTGGAGACA; (R):CACACACGCACCTCAAAG
Transcription Factors	*Krüppel-like factor 4 (klf4)*	NM_001142713	(F):GCATACGGCAGCAGCAACTT; (R):GCTCGGCTACCAGACTGTGT
	*krüppel-like factor 11 (klf11)*	BT043977	(F):CGCTCTCCTCCTTGTGTATGA; (R):TGTGGATGGCAGTGGTAGA
	*krüppel-like factor 2 (klf2)*	BT059519	(F):GGTTGTGGCTGGAAGTTC; (R):CAGTGTGCTTACGGAAATGG
Inflammation	*arachidonate 5-lipoxygenase (lox5)*	NM_001139832	(F):ATCCACCAGACAGTCACACACCTTC; (R):GCCACTCCAAACACCTCCGAGAC

The mitogen-activated protein kinases (MAPK) family includes extracellular signal-regulated kinases (ERK), p38 and c-Jun N-terminal kinases. Sequences previously described in Atlantic salmon were identified for *erk1* (*mapk3*), *p38a*, *p38b1* and *p38b2*. Predicted Atlantic salmon sequences for *erk2* (*mapk1*), *erk3* (*mapk6*) and *jnk1* (*mapk8*) were detected from BLAST searches using characterised sequences from zebrafish. Primers were designed for all seven *mapk* genes, but based on Ct values and analysis of melting curves in a preliminary PCR analysis (data not shown), only *p38b* (primers designed to detect both *p38b1* and *p38b2*), *erk3* and *jnk1* were consistently expressed in gills and thus selected for gene expression analysis. Atlantic salmon sequences were unequivocally found for all other genes used in this study and their expression in gills was also confirmed by a preliminary PCR analysis.

### Gene expression profile

#### Global assessment: Principal component analysis

**Figure 5:**
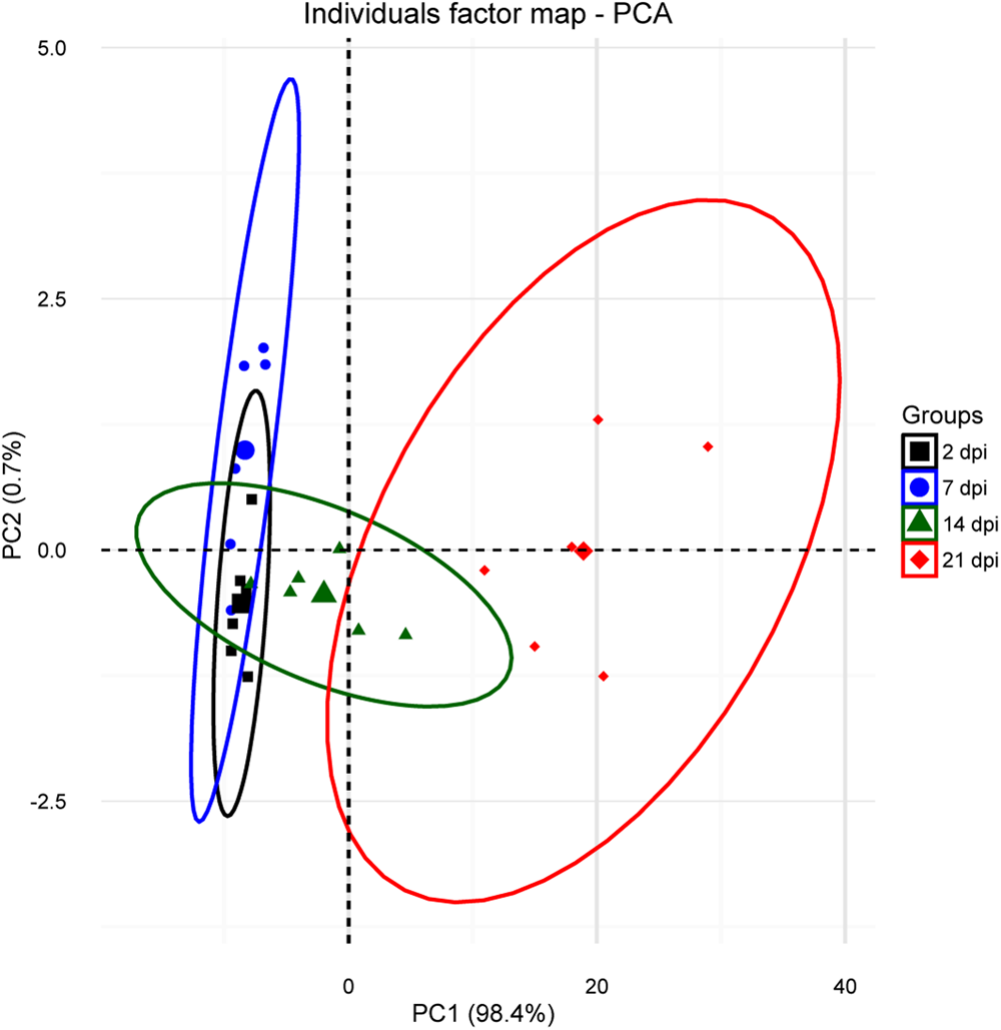
PCA analysis for the *in vivo* challenge samples representing the distribution of infected samples at 2, 7, 14 and 21 dpi. Analysis was based on fold-changes of all genes for each individual sample (smaller symbols) relative to the negative control at each time point. The ellipses indicate the group dispersion/variability from the centroid (larger symbols) calculated using all individual fold-changes values/group.

To obtain an overview of the expression profiles of the different groups tested we performed exploratory data analyses using principal component analysis (PCA). The PCA analysis of the expression profile of the 21 selected genes in gills for the challenge samples (Fig. 5) showed that samples taken at the early stages of infection (2 and 7 dpi) were very similar and with low variability. Consecutive samples (14 and 21 dpi) displayed an increasing deviation along the principal component 1 (PC1) that contributed to most (98.4%) of the observed variation. Samples taken at 14 and 21 dpi formed clearly distinct clusters, and variability among individual samples within groups increased with disease severity.

**Figure 6:**
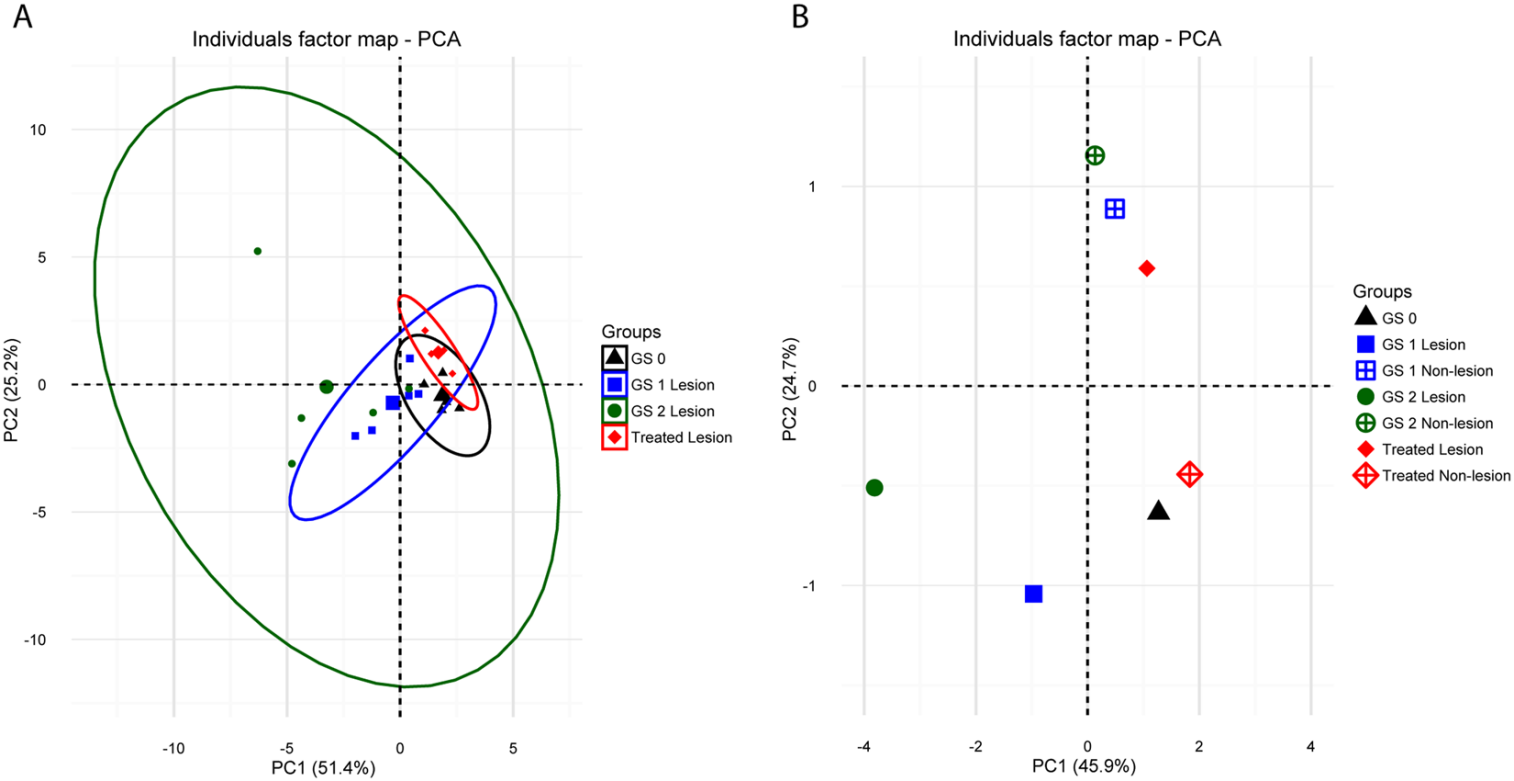
(**A**) PCA analysis for the field samples representing the distribution of lesion samples of GS 1, GS 2 and treated groups. Analysis was based on fold-changes of all genes for each individual sample (smaller symbols) relative to GS 0. The ellipses indicate the group dispersion/variability from the centroid (larger symbols) calculated using all individual fold-changes values. (**B**) PCA analysis for the field samples representing the distribution of lesion and non-lesion samples of GS 1, GS 2 and treated groups. Analysis is based on fold-changes of all genes for each individual sample relative to GS 0. For clarity, individual values were omitted from the plot and only the group centroids were plotted.

The PCA analysis for the field samples, performed with fold-changes for lesion areas at GS 1, GS 2, and treated groups, relative to GS 0 (Fig. 6A), showed a shift along PC1 that correlated with the disease severity. Upon treatment, a reversion to control values could be observed on PC1. Similar to the results obtained for the *in vivo* challenge, variability among individual samples within groups again increased with disease severity. PC1 and PC2 contributed to 51.4% and 25.2% of the observed variability, respectively. An additional PCA analysis was performed using fold-changes of all samples (lesion and non-lesion areas of all sampling points) in relation to GS 0 (Fig. 6B). This analysis revealed that non-lesion areas of GS 1 and GS 2 and treated groups were very close to GS 0 along the PC1 (45.9% of the observed variability) and separated from lesion areas on both PC1 and PC2 (24.7% of the observed variability). Interestingly, lesion-treated samples were very close to the non-lesion GS 1 and GS 2 groups, while non-lesion treated samples were closer to GS 0.

When exploring into more detail the factors that contributed most to PC1 and PC2 in the PCA analyses of Figs 5 and 6, it was determined that most of the variability was driven by the expression of mucins, *il4/13a*, *il4/13b2* and *tnfα3*. The contribution values for all the genes studied can be found in Supplementary Table S1. In order to assess whether changes in these genes were related to disease severity, partial least squares regression (PLSR) was performed using the matrix of expression values of mucins, *il4/13a*, *il4/13b2* and *tnfα3* as the X variable, and the gill score as Y variable. The results from the controlled *in vivo* challenge experiment were used as the training set to construct the model to predict the gill scores of the field experiment. The results of the prediction are shown in Table 2 and clearly show a correlation of the expression of these genes with the severity of the infection (i.e. GS) in lesion areas. On the other hand, as already seen in the PCA analyses, the model predicts gill scores closer to the control value (GS 0) for the non-lesion and treated groups.

**Table 2:** Field experiment gill score values predicted using partial least squares regression.

Group	GS 0	GS 1 Lesion	GS 1 Non-lesion	GS 2 Lesion	GS 2 Non-lesion	Treated Lesion	Treated Non-lesion
Predicted GS	0.14 ± 0.078	1.03 ± 0.419	−0.01 ± 0.089	2.09 ± 0.519	0.03 ± 0.102	−0.19 ± 0.029	0.05 ± 0.061

The model was constructed using the expression values of the genes that contributed most to the variability observed in components 1 and 2 of the PCA analyses shown in Figs 5 and 6 (*muc5*, *muc18*, *muc2*, *il4/13a*, *il4/13b2* and *tnfα3*) as X variables, and gill score (GS) as Y variable. The *in vivo* challenge dataset served as the training set. Values show the mean (±SEM) predicted GS of the different field experiment sample groups.

#### Detailed assessment: Gene expression analysis

**Figure 7:**
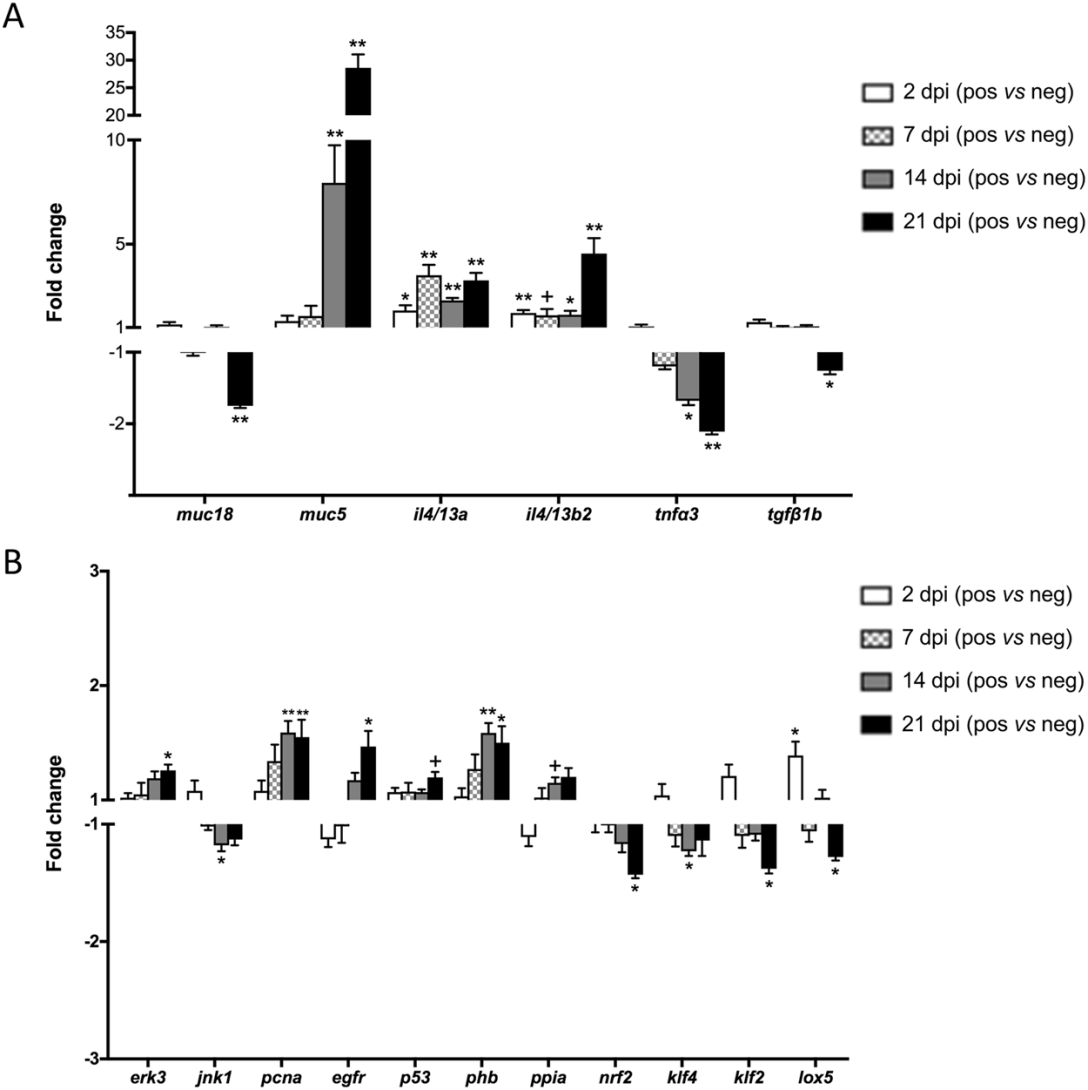
Relative expression of genes showing statistically significant changes (^+^p < 0.1, ^*^p < 0.05, ^**^p < 0.01) in the *in vivo* challenge experiment. Mucins and cytokines are represented in (**A**), and the other differentially expressed genes are shown in (**B**). Fold changes (FC) were calculated as the relative expression in the infected (positive) *versus* the non-infected (negative) samples. Genes showing FC > 1 were up-regulated in infected samples, whereas genes with FC < 1 were down-regulated in infected samples.

**Figure 8:**
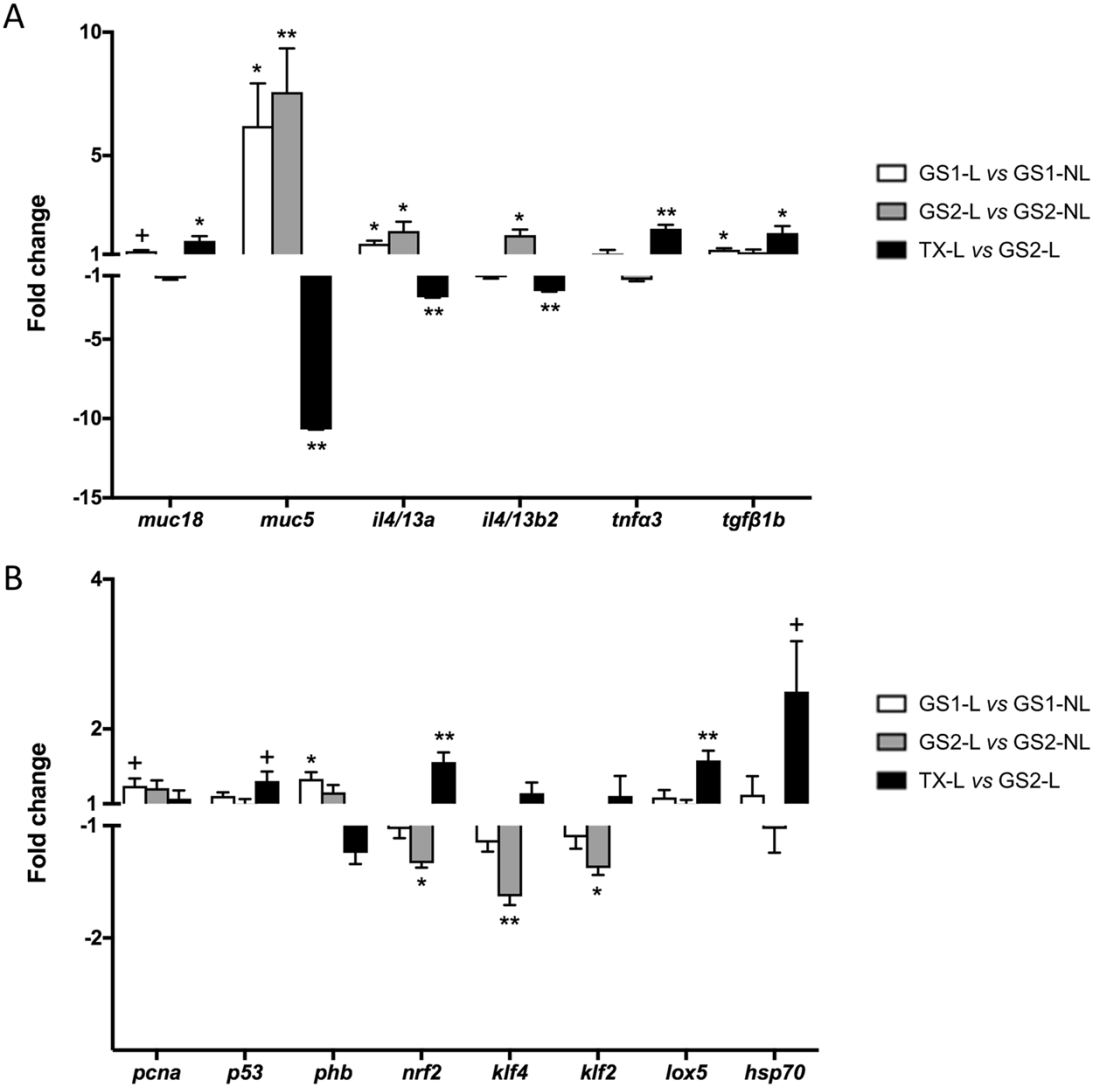
Relative expression of genes showing statistically significant changes (^+^p < 0.1, ^*^p < 0.05, ^**^p < 0.01) in the field samples. Mucin and cytokines are represented in (**A**), and the other differentially expressed genes are shown in (**B**). Fold changes (FC) were calculated as the relative expression in lesion (L) *versus* non-lesion (NL) areas for GS 1 and GS 2, and in lesion areas post-treatment (TX-L) *versus* lesion areas at GS 2. Genes showing FC > 1 were up-regulated in lesion areas or post-treatment, whereas genes with FC < 1 were down-regulated in lesion areas or post-treatment.

The results of the exploratory data analyses clearly showed that changes related to disease severity were taking place and that freshwater treatment was able to revert, at least partially, to the control phenotype. Therefore, we proceeded to study these changes in detail in order to further characterize the mechanisms involved in AGD progression. The relative mRNA expression of the 21 studied genes for all individual samples are shown in Supplementary Table S2 (*in vivo* challenge) and Supplementary Table S3 (field samples). From the 21 genes analysed, 17 genes in the *in vivo* challenge and 14 genes in the field sampling showed statistically significant different expression in at least one of the sampling points. Of note, some genes were so consistently expressed that they showed statistical significance differences despite showing low fold change values (i.e. *jnk1* or *nrf2*). To simplify the visualization of the results, only fold changes (FC) of the differentially regulated genes are represented in Fig. 7 (*in vivo* challenge) and Fig. 8 (field samples).

In both trials, mucins (especially *muc5*) and cytokines (particularly *il4/13a* and *il4/13b2*) showed the most substantial and consistent changes between infected and non-infected or treated samples, and between lesion and non-lesion areas. *muc5* was progressively and substantially up-regulated at 14 (FC = 7.94) and 21 (FC = 28.61) dpi, and in lesion areas at GS 1 (FC = 6.20) and GS 2 (FC = 7.57), whereas *muc18* was down-regulated at 21 dpi. Both mucins showed a reversed trend post-treatment, with down-regulation of *muc5* and up-regulation of *muc18* in treated lesions versus GS 2 lesions. For the immune genes, *il4/13a* and *il4/13b2* were significantly up-regulated in infected fish at all sampling points and in lesion areas of GS 1 and GS 2 (except for *il4/13b*2 at GS 1), whereas *tnfα*3 showed a progressive down-regulation from 7 to 21 dpi and *tgfß1b* was down-regulated at 21 dpi. As observed for the mucin genes, reversed and significant fold changes were detected for all cytokines tested after treatment. *muc2* was detected in gills, but the expression was very low and variable between individuals. No significant differences were detected in relation to disease progression, but interestingly, *muc2* expression in the *in vivo* challenge samples was detected in all individuals except in three out of the six individuals from the infected group at 21 dpi (Supplementary Table S2).

Expression of *mapk* and *p38b* genes did not show significant changes in the field samples, but *erk3* and *jnk1* were significantly up- and down-regulated, respectively, at clinical stages of infection in the *in vivo* challenge. Krüppel-like factors *klf2* and *klf4* were consistently down-regulated in clinical AGD and AGD lesions in the *in vivo* challenge and field samples, and showed a slight up-regulation post-treatment. No changes were detected for *klf11*.

In the *in vivo* challenge samples, significant up-regulation at 14 and/or 21 dpi was detected for *pcna*, *egfr*, *p53*, *phb* and *ppia*, while *nrf2* was down-regulated, and *lox5* showed up- and down-regulation at 2 and 21 dpi, respectively. Fewer changes were detected in the field samples. *pcna* and *phb* were up-regulated in lesion areas at GS 1, and *p53* was up-regulated post-treatment. Treatment resulted in a shift on *nrf2* and *lox5* expression, with up-regulation of both genes after treatment. *hsp70* showed no changes during the *in vivo* challenge and in the field samples *hsp70* only appeared significantly up-regulated after treatment.

## Discussion

In the current study, we investigated the expression of a panel of selected genes to define their involvement in Atlantic salmon gills during AGD. Being the hallmarks of AGD increased production of mucus and hyperplasia of the lamellar epithelium, we specifically targeted genes that could be directly related to these pathways. The study was performed using samples from an experimental infection with controlled parameters, but also from a natural outbreak in a farm, including a sampling point post-freshwater treatment to eliminate the amoebae. In the field of fish diseases, most gene expression studies are performed using experimental samples, but field samples are also required in order to validate the results obtained under laboratory conditions. The results from both trials were very consistent and underlined the importance of mucins and Th2 cytokines in AGD. Indeed, the predictive model constructed, based on the expression values of these genes from the experimental infection, was able to accurately predict the disease severity of the field samples, exposing the experimental infection model as a reliable tool to study this disease under controlled conditions. To our knowledge, this is the first report on Atlantic salmon gill mucins regulation upon gill disease, and pose an important starting point for future characterizations of the poorly known fish mucins.

PCA of the *in vivo* challenge and field samples showed consistent clustering of the different sampling groups, and an increasing separation of groups upon disease severity. Thus, the expression profile of the selected genes provided a good measure on the advancement of the disease. In fact, out of the 21 genes in the array, 17 and 14 genes were significantly regulated in the *in vivo* and field experiments, respectively. In addition, PCA results of field samples highlighted the multifocal distribution of the gill lesions, with the highest variability in lesion areas. Lesion-specific regulation was previously described in genes involved in immunity^26,27^ and antigen processing and presentation^30^. Interestingly, gills were able to partially revert the changes in the expression of the selected genes as rapidly as four days after freshwater treatment and, as expected, the reversion to control levels was almost complete in non-lesion areas. Even though the experimental design and analysis differed between trials, gene expression results from challenge and field samples showed similar and comparable trends, and in both scenarios changes in mucins and cytokines accounted for most of the variability observed between groups, highlighting their key role in AGD.

Two different mucin sequences, *muc5* (secreted and gel-forming) and *muc18* (membrane-bound), were consistently detected in gills of Atlantic salmon. *muc5* was substantially up-regulated in fish displaying clinical AGD, and in AGD lesions relative to non-lesion areas; whereas *muc18* was significantly down-regulated at 21 dpi. Secreted mucins are responsible for the rheological properties of mucus, and *muc5* is most likely the main component of the characteristic mucus patches comprising AGD lesions. A sequence characterised as *muc2* was also detected in gills, but the expression was very low and variable among individuals. Muc2 is the major secretory mucin in intestine of mammals and fish^16,18^. Muc2 has also been detected in mammalian respiratory epithelium, but, similar to the results in gills, its expression in lung is in some cases weak or undetectable^38^. These results indicate a key role of Muc5-type mucins in respiratory organs of fish, as is the case in mammals, and show the potential of *muc5* as biomarker for AGD. Indeed, MUC5AC and MUC5B are the principal components of airway mucus in humans^39^, and overexpression of one or both types is a hallmark of different airway diseases^15,40^. In mammals, birds and reptiles, type 5 mucins are divided into Muc5AC and Muc5B, but this classification is not so clear in fish and amphibians^17,41^. This complexity and unclear classification was evidenced in the phylogenetic analysis, where the Atlantic salmon *muc5* sequence grouped together with both mucins described as Muc5AC and Muc5B. Sveen *et al*.^21^ reported seven genes of secreted mucins in Atlantic salmon (i.e. *muc5ac.1*, *muc5ac.2*, *muc5ac.3*, *muc5ac.4*, *muc5b*, *muc2.1*, *muc2.2*), of which, *muc5* genes were detected in gills, skin and pyloric caeca (genes labelled as *muc5ac.2* and *muc5ac.4* being the highest expressed in gills), whereas *muc2* genes showed highest expression in intestine and pyloric caeca. In their study, Sveen *et al*.^21^ showed upregulation of *muc5ac.2/4* and *muc2.1/2* in gills after handling stress, but the FC for *muc5ac.2/4* (i.e. FC = 0.8) was significantly lower than the one reported here for *muc5* upon parasitic infection. In zebrafish, six partial *muc5*-type sequences have been identified (labelled *muc5.1* to *muc5.6*), of which *muc5.1* and *muc5.2* are detected in gills, but only *muc5.1* in lamellae^16,17^. Mucin sequences described as *muc5B* in common carp^19^ and *muc5AC* in channel catfish (*Ictalurus punctatus*)^42^ have also been detected in gills. Mucus properties and composition vary among fish species and can change depending on disease and environmental factors^43,44^. In a previous study, the increase in goblet cells numbers in AGD lesions was found to be predominantly due to an increase in the number of cells containing neutral mucins, although a slight increase in cells containing carboxylated mucins was also observed^45^. In another study, Roberts & Powell^44^ detected decreased viscosity and increased glucose and protein concentration in skin mucus of AGD affected fish.

Gill diseases can be caused by numerous agents, but morphologic patterns of tissue reaction are limited. Comparative pathology with other respiratory disorders can provide valuable insights into pathogenic mechanisms and potential therapeutic targets. Many chronic inflammatory airway diseases in mammals involve mucus hypersecretion, and different stimuli and signaling pathways have been investigated in relation to mucin overproduction and goblet cell hyperplasia and metaplasia. The mechanisms underlying mucus hypersecretion in fish have been less explored, but interspecies extrapolation may be possible based on conserved gene functions. Th2 cytokines (mostly IL13) in mammals are known to stimulate goblet cell hyperplasia and overexpression of Muc5AC and/or Muc5B^32,33,46^, and the distinct pattern of *muc5* and *il4/13a* and *il4/13b2* expression detected in AGD may indicate a similar mechanism in fish. Benedicenti *et al*.^23^ reported up-regulation of the fish homologues *il4/13a* and *il4/13b2* and down-regulation of Th1, Th17 and Treg markers in late stage AGD. The present study showed up-regulation of *il4/13a* and *il4/13b2* in infected fish as early as 2 dpi and they were particularly over-expressed in lesion areas. This demonstrates the rapid and lesion-specific response of gills to the parasite and the importance of *il4/13a* and *il4/13b2* on AGD pathogenesis. Based on the results obtained for the different sampling points in both trials, up-regulation of *il4/13a* may appear slightly more consistent and specific to the infection than *il4/13b2*.

EGFR promotes cell proliferation, differentiation and migration, but it is also a key target leading to goblet cell hyperplasia/metaplasia^34,47^ and MUC5AC expression^35,48^. EGFR has been related to IL13-induced epithelial proliferation^49^ and mucin production^50^ in mammals. TNFα can also stimulate EGFR expression, but no changes in *tnfα1* or *tnfα2* were detected in previous AGD investigations^23,24,51^ and *tnfα3* in the present and a previous^23^ study was significantly down-regulated in clinical AGD. TNFα is one of the main acute pro-inflammatory cytokines, and the decreased expression of *tnfα3* could be interpreted as an impaired immune response to the disease. However, TNFα and TGFβ can also induce growth arrest and/or apoptosis^52,53^, and therefore their down-regulation is probably more related to sustaining the prominent cell proliferation occurring in AGD. LOX5 has a central role in the biosynthesis of leukotrienes, inflammatory mediators with important roles in allergy and innate and adaptive immunity^54–56^. The expression profile of *lox5* observed during *N. perurans* infection showed an initial increase at 2 dpi, suggesting an early triggering of the leukotriene pathway. However, at 21 dpi, the expression of *lox5* was significantly down-regulated suggesting an impaired host response or, more likely, a specific regulation of this pathway at later time points. In fact, in mammals IL4/IL13 can inhibit *LOX5* and concomitantly up-regulate *LOX15* expression in dendritic cells^57^.

Muc18, also named melanoma cell adhesion molecule, belongs to the immunoglobulin superfamily. *muc18* was the most abundant mucin detected in gills of gilthead sea bream^18^ and, in accordance, its relative expression in the present study was much higher than that of *muc5* (Supplementary Tables S2 and S3). Muc18 is involved in cell-cell interactions and cell migration, and altered expression can affect cell motility and invasiveness^58^; reason why Muc18 has been studied in tumorigenesis and reported to be up- or down-regulated depending on the type of cancer^59–61^. Pro-inflammatory roles of Muc18 have also been described^62^, and its expression is increased in airway inflammation^63,64^. The role of this abundant membrane-bound mucin in gills and the stability of the glycocalyx remains to be investigated.

Krüppel-like factors (KLFs) and mitogen activated protein kinases (MAPKs) are involved in pathways that regulate cell proliferation, differentiation, stress response and apoptosis^36,37^. KLFs are downstream signals of MAPKs, and their expression was investigated to assess potential mechanisms involved in AGD epithelial hyperplasia. All main members of the MAPK family have been identified in fish^65^, but limited data exist on *mapk* expression during infectious diseases in fish. Three important representatives of the MAPK family (*erk3*, *jnk1* and *p38b*) were included in this study. MAPKs pathways are well conserved across vertebrates and the MAPK family is divided into four main cascades: the extracellular signal-regulated kinase 1/2 (ERK1/2), the c-Jun N-terminal kinase (JNK), the p38, and the ERK5 cascade. Largely, JNK and p38 signaling pathways are activated by pro-inflammatory cytokines, including IL1ß, TNFα and IL6, or in response to cellular stress, leading to inflammation, cell cycle arrest and/or apoptosis; while ERK1/2 is mostly activated by mitogenic factors and growth factors to promote cell proliferation and prevent apoptosis^36,66,67^. ERK3 is considered an atypical MAPK with unclear functions to date, but there are reports of increased ERK3 expression associated with cell proliferation^68^. *erk3* and *jnk1* changes detected in the present study were small but consistent with a cell proliferation process. It is important to note that activation of MAPKs is induced by phosphorylation. Therefore, gene expression analyses, while providing a hint on the pathways involved, might not be enough to completely define the contribution of these proteins in AGD progression and resolution.

KLFs are zinc finger-containing transcription factors and alterations in their expression and function have been associated with several human diseases^37^. Information on fish KLFs is very scarce, but interest exists on the conservation and function of the homologues proteins in lower vertebrates^69^. In mammals, KLF2 and KLF4 are highly expressed in lung and gastrointestinal tract, respectively^70^. In the present study, the relative expression of *klf2* was considerably higher than that of *klf4* (Supplementary Tables S2 and S3), which indicates a potential conservation and association of KLF2 with respiratory organs. KLFs can function as cell cycle activators or repressors in a cell type- and promoter-dependent manner, but KLFs are emerging as potential tumour suppressor genes and down-regulation of both KLF2 and KLF4 has been associated with several cancer types^71–74^. The significant down-regulation of *klf2* and *klf4* detected in both *in vivo* challenge and field samples, indicates a function in cell cycle arrest of these genes in AGD. KLF2 is also involved in immunity and suppression of KLF2 in dendritic cells has been reported to amplify Th2 responses^75^. In addition, KLF2 has been described to inhibit pro-inflammatory activation of monocytes in mammals^76^ and in fish^77^. Lu *et al*.^77^ showed that Klf2 knockdown in *Plecoglossus altivelis* monocytes/macrophages resulted in the enhancement of cytokine production (Il1β, Tnfα), phagocytotic and bactericidal capability after *Listonella anguillarum* exposure. On the other hand, KLF4 is associated with goblet cell differentiation in the gastro-intestinal tract^78^, and a similar role has been reported in zebrafish^79^.

Down-regulation of tumour suppressor p53 (*p53*) has been suggested as one of the mechanisms underlying cell proliferation in AGD^28^, but this pattern was not observed in the current study. This differing result shows the value of multiple studies and the use of field samples, as several factors, including infection dose, sampling times, fish size, and previous disease status, among others, may affect gene expression results. On the other hand, *p53* appeared up-regulated after freshwater treatment, which suggests its involvement in cell death and tissue remodelling post-treatment. NRF2 regulates the expression of antioxidants and its down-regulation in challenge and field samples is indicative of oxidative stress, which has previously been associated with AGD pathogenesis^29,80^. Moreover, NRF2 deficiency can exacerbate airway injury and inflammation in mammals^81–83^. Phb, Ppia and Hsp70 protein levels were significantly increased in AGD affected fish in a former proteomic study^31^. Phb and Ppia are related to inflammation, cell proliferation and inhibition of oxidative stress induced apoptosis. Consistently, *phb* was up-regulated in challenge and field samples. In our former proteomic study^31^, with an experimental setup comparabale to the one used for the current challenge experiment, Phb showed 1.2 and 1.6 fold change increase after 14 and 21 dpi, respectively. These values are consistent with the present results of the challenge experiment where *phb* was also significantly up-regulated at 14 and 21 dpi with FC values of 1.59 and 1.50, respectively. On the other hand, the proteomic study showed significant up-regulation of Ppia after 2, 7 and 14 dpi with FC values of 1.3, 1.3 and 1.5, respectively, whereas, in the current study up-regulation of *ppia* was mostly not significant. Protein abundance of Hsp70 did not correlate with gene expression, and *hsp70* was only up-regulated post-treatment.

In conclusion, AGD progression was characterised by the up-regulation of the Th2 IL4/13 cytokines, and genes related to mucin secretion and cell proliferation, while a number of Th1 cytokines and pro-apoptotic genes were down-regulated. Currently used treatments against AGD aim to remove excessive gill mucus and eliminate pathogenic amoebae. Gills hold an extraordinary plasticity and repair capacity, and the results obtained post-treatment in the present study show that freshwater bath is not only effective against the parasite, but also supports the partial restoration of the host immune, mucosal, structural and anti-oxidant status. An increased rate of apoptosis and supressed rate of mitosis are most likely involved in the gill remodelling occurring post-treatment. The current results also show the activation of Th2 cytokines as early as 2 dpi, and suggest an association of *il4/13a* and *il4/13b2* expression with *muc5* expression and secretion. In mammals, goblet cell hyperplasia and increased mucus secretion mediated by Th2 immune responses (IL13 and IL4) are known mechanisms for certain parasitoses such as infections with helminth parasites *Nippostrongylus brasiliensis* and *Trichinella spiralis*^84,85^. In AGD pathogenesis, the type 2 response characterised by higher expression of *il4/13a*, *il4/13b2*^23^ and *arg2b*^86^ has been proposed to be related with activation of alternative (M2) macrophages. M2 macrophages already showed to be crucial, particularly due to their important tissue repair function, against helmint parasites infections^87^. Thus, the presence and activation of M2 macrophages during AGD should be further studied. Mucus hypersecretion and cellular hyperplasia go hand in hand during AGD, and both changes are restricted to lesion areas with the presence of amoebae. Interactions and common mechanisms are therefore likely; e.g. Th2 cytokines have been associated with epithelial proliferation and anti-apoptotic mechanisms^88–90^, and aberrant mucin expression occurs in certain types of cancer^38^.

This is the first study exploring expression of gill mucins in Atlantic salmon during an infectious process. The results show the importance of *muc5* in AGD pathology and severity, and prove the effectiveness of freshwater treatment to reestablish gill homeostasis under field conditions. Gill diseases are currently one of the main health challenges for the industry, but significant knowledge gaps still exist on gill mucosal immunity and respiratory pathogenesis. Further research on *muc5* sequences and their differential expression in different gill conditions of Atlantic salmon and other fish species would contribute to improve our understanding of gill health.

## Materials and Methods

All methods described were carried out in accordance with relevant guidelines and regulations. All experimental protocols were approved by The Health Products Regulatory Authority (HPRA) in Ireland under project authorisation number AE19114/P001, and following the Animals Scientific Procedures Act 1986 (Directive 2010/63/EU transposed into Irish law by S.I. No 543 of 2012).

### *In vivo* challenge

Samples for this study were obtained from a previous challenge described in Marcos-López *et al*.^31^. Figure 1 summarizes the details of the *in vivo* challenge.

#### N. perurans isolation and experimental bath challenge

Gill mucus containing *N. perurans* was collected from naturally infected Atlantic salmon (displaying clinical AGD) from a commercial farm in the west of Ireland. Infected mucus was transferred into filtered sterile sea water, and the suspended solution plated into marine malt yeast agar plates. The amoeba culture (Fig. 2A) was maintained at 18 °C and species identification was confirmed by real-time polymerase chain reaction (PCR), as described by Downes *et al*.^91^. The experimental challenge was carried out at the Daithi O’Murchu Marine Research Station (Bantry, Co. Cork, Ireland). Naïve Atlantic salmon smolts (~85 g; n = 300) were distributed into four circular 1,000 L tanks (n = 75) and left to acclimatise for 10 days. Two tanks were challenged with 1,800 amoebae/L (infected tanks), whereas the other remaining two tanks were kept as negative controls (non-infected tanks). Prior to the inoculation with *N. perurans*, the water level of all tanks, including the control ones, was lowered to 300 L. Following inoculation, all tanks were maintained for 4 h at 300 L and subsequently refilled to 1,000 L. Water temperature ranged from 10.5 °C to 11.5 °C and salinity was 35 practical salinity units (PSU). Fish were fed a commercial salmon diet at 1% body weight per day.

#### Collection of samples

Sampling points were set at 2, 7, 14 and 21 days post-infection (dpi). At each sampling point, 10 fish per tank were anaesthetised with 100 mg L^−1^ of tricaine methanesulfonate (Tricaine, Pharmaq), and gills scored as per the standard protocol used in Atlantic salmon aquaculture. Gill scoring, adapted from Taylor *et al*.^92^, is a visual scoring system entailing a macroscopic examination of all gill arches (Fig. 2B) of each individual fish for the presence and extent of AGD lesions. The severity of the macroscopic lesions ranges from 0 (non-affected) to 5 (severely affected). Out of the 10 fish examined per tank at each time point, 3 fish were euthanized with an overdose of anaesthetic (400 mg L^−1^ of tricaine methanesulfonate) and gill samples excised and placed into RNAlater^TM^ (Ambion) for gene expression analysis. Gill samples were taken from the 2^nd^ right gill arch. In AGD positive fish showing no AGD lesions in the 2^nd^ right gill arch, samples were taken from adjacent arches to ensure inclusion of lesion areas. Samples were stored at −80 °C until use. The remaining seven fish per tank were allowed to recover in clean oxygenated seawater and returned to the trial tanks. In addition, histology samples and gill swabs for *N. perurans* PCR^91^ were taken to confirm AGD and presence of *N. perurans*. For histological analyses, samples were fixed in 10% neutral buffered formalin, routinely processed, embedded in paraffin wax blocks, sectioned (3–5 μm), and stained with haematoxylin and eosin (H&E).

### Field sampling

Samples for this study were obtained from a previous sampling described in Marcos-López *et al*.^93^. A summary of the details for the field sampling can be found in Fig. 1.

#### Fish and environmental conditions

Atlantic salmon S1 smolts (average initial weight 67 g, initial stocking density 0.55 kg m^−3^) in a commercial farm in north west Ireland were monitored from sea transfer (29^th^ March 2016) until development of first gross signs of AGD (80 days post-transfer (dpt)) and subsequent freshwater bath treatment (<3 practical salinity units (PSU) for 3 h) at 108 dpt. Gill scoring was conducted on a weekly basis, and sampling points were set at gill score (GS) 0 (fish showing no gross AGD lesions), GS 1 (early AGD), GS 2 (advanced AGD), and 4 days after freshwater treatment. Accordingly, sampling points took place at 21 dpt (GS 0), 85 dpt (GS 1), 92 dpt (GS 2), and 112 dpt (4 days post freshwater treatment). Average weights for the four sequential sampling points were 85.2 g, 282.0 g, 312.1 g and 375.3 g, and water temperature was 9 °C, 14.9 °C, 15.0 °C and 15.2 °C, respectively. Salinity was 32 to 33 PSU through the entire monitoring period, and fish were fed a commercial salmon diet at 2% body weight per day.

#### Collection of samples

At each sampling point, 30 randomly caught fish from a single pen (same pen sampled in all time points) were anaesthetised with 100 mg L^−1^ of tricaine methanesulfonate and gills scored as described for the *in vivo* challenge. Five fish showing equal GS were further euthanized with an overdose of anaesthetic (400 mg L^−1^ of tricaine methanesulfonate), and gill samples excised and stored as described for the *in vivo* challenge. Separate samples comprising gill areas with and without AGD lesions were taken for GS 1 and GS 2 and post-treatment. As defined for the *in vivo* challenge, gill swabs for PCR and histology samples were taken to confirm infection and disease.

### Gene sequence analysis and primer design

The panel of genes selected for this study included mucins (*muc18*, *muc5*, *muc2*), mitogen-activated protein kinases (*mapk*), cytokines (*tnf*α3, *tgfβ1b*, *il4/13a*, *il4/13b2*), an oxidative stress marker (nuclear factor erythroid 2-related factor 2 (*nrf2*)), proteins previously detected to be differentially expressed in AGD in a proteomic study^31^ (prohibitin (*phb*), peptidylprolyl isomerase (*ppia*), heat shock 70 kDa protein (*hsp70*)), markers of cell proliferation (proliferating cell nuclear antigen (*pcna*), epidermal growth factor receptor (*egfr*)), a marker of apoptosis (cellular tumour antigen p53 (*p53*)), Krüppel-like factors (*klf4, klf11, klf2*), and a marker of inflammation (arachidonate 5-lipoxygenase (*lox5*)).

The sequence for mucin 5 (*muc5*) was obtained from an isotig sequence (isotig 05987) reported in Atlantic salmon skin^22^. Other mucin sequences were identified by BLAST queries (E-value < 4e^−75^) using mucin sequences reported in other fish species (*Sparus aurata*^18^; *Cyprinus carpio*^19^; *Danio rerio*, http://www.medkem.gu.se/mucinbiology/databases/db/Mucin-zebrafish-2015.htm). Sequences for the other selected genes were obtained from published data in Atlantic salmon, term-searches in GenBank for automatically annotated genes, and BLAST queries using sequences described for other fish species. Primers were designed to obtain amplicons of 50–150 bp in length using Beacon designer 7.60. See Table 1 for a list of selected genes, accession numbers and primer sequences.

Sequence alignments and phylogenetic analysis of mucin sequences analysed in Atlantic salmon gills in this study (*muc5, muc18, muc2*) and 17 other mucin sequences from 10 different species were carried out in MEGA version 6^94^. A phylogenetic tree was constructed using the Neighbor Joining method with the Poisson model, and reliability of the tree was assessed by bootstrapping, using 1,000 bootstrap replications.

### Gene expression analysis

Total RNA from gills was extracted with a MagMAX-96 for microarray kit (Life Technologies) after tissue homogenization in TRI reagent following manufacturers’ instructions. RNA quantity and purity was determined by Nanodrop (Thermo Scientific) with absorbance ratios at 260 nm/280 nm above 1.9. Reverse transcription (RT) of 500 ng of total RNA was performed with random decamers, using the High-Capacity cDNA Archive Kit (Applied Biosystems) following manufacturers’ instructions. RT reactions were incubated for 10 min at 25 °C and 2 h at 37 °C. Negative control reactions were run without reverse transcriptase. During the design of the array, at least two sets of primers were designed for all the targeted molecules and pathways. All primer sequences were blasted against the available databases to rule out possible amplification of similar undesired products. Specificity and performance of each pair of primers was assessed by analysis of melting curves and linearity of serial dilutions of RT reactions. PCR product presence and sizes were checked in a 1% agarose gel. Only primers for genes with clear expression in gill tissue (Ct values of undiluted samples <30 and presence of a clear band in the gel), and all with similar efficiencies and between 90 and 98% were used in the study. Real-time quantitative PCR was carried out with a Mastercycler Ep Realplex real-time PCR system (Eppendorf) using a 96-well PCR array layout designed for the simultaneously profiling of a panel of 21 genes. Each PCR-well contained 12.5 µl SYBR Green Master Mix (Bio-Rad), 5 µl of specific primers at a final concentration of 0.9 µM, and 7.5 µl diluted RT reaction. PCR amplification included an initial denaturation step at 95 °C for 3 min, followed by 40 cycles of denaturation for 15 s at 95 °C and annealing/extension for 60 s at 60 °C. The specificity of reactions was verified by analysis of melting curves. Controls for general PCR performance were included on each array, and all the pipetting operations were performed with the EpMotion 5070 Liquid Handling Robot (Eppendorf). The efficiencies of all PCR runs were always higher than 90% (ranging from 91 to 99%). Data acquired during the PCR extension phase were normalised using the delta-delta Ct method^95^. Four potential housekeeping genes (*β-actin*, *elongation factor 1α*, *α-tubulin* and *18S rRNA*) were previously tested for stability using the GeNorm software. The most stable reference gene among conditions was *β-actin* and it was used in the normalization procedure. For multi-gene analysis comparisons, relative gene expression was referenced to the expression level of *egfr* of control fish (negative control at 2 dpi for the challenge samples and GS 0 for the field samples) with an arbitrarily assigned value of 1.

### Statistical analysis

Principal component analyses (PCA) were performed using fold-changes of all individual samples relative to the negative control at each time point for the *in vivo* challenge or relative to GS 0 for the field experiment. The default *prcomp* function of the R statistical software (v3.0.2) was used to calculate the principal components and visualizations were constructed using the *factoextra* (v1.0.4) and *gplots* (v3.0.1) R packages. Ellipses in the PCA graphs are confidence ellipses with a confidence level of 0.95 and the centroids represent the center of the mass of the points per group. Partial least squares regression (PLSR) was performed using the *pls* (v2.6.0) R package. The model was constructed using the expression values of *muc5*, *muc2*, *muc18*, *il4/13a*, *il4/13b2* and *tnfα3* (selected due to their high contribution in the PCA analyses) as X variables, and gill scores (GS) as the Y variable. The data from the controlled *in vivo* challenge experiment was used to train the model. The best performing model in the training dataset cross-validation (LOO or leave-one-out cross-validation) was the one using 3 components (root mean square error, RMSE = 0.59). This model was used to predict the GS of the field experiment (RMSE = 0.51). Gene expression data were represented as fold change (FC) ± SEM. FC for the challenge samples were calculated using the relative expression of the AGD positive samples versus the negative control at each time point. FC for the field samples were calculated using relative expression in lesion versus non-lesion areas for GS 1 and GS 2 to assess specificity/distribution of changes during clinical AGD, and using relative expression in lesion areas post-treatment versus lesion areas at GS 2 to assess treatment effects. Statistical analyses and graphs were performed using GraphPad PRISM v7.0. Differences were evaluated using the Student’s *t*-test, after checking that the data were normally distributed, and the significance level was 95% (p < 0.05) unless otherwise stated.

## Electronic supplementary material

Supplementary Tables

## Data Availability

All data supporting this study is included in the results section of the manuscript, the supplementary material or openly available in public databases.

## References

[1] Gjessing MC, Thoen E, Tengs T, Skotheim SA, Dale OB (2017). Salmon gill poxvirus, a recently characterized infectious agent of multifactorial gill disease in freshwater- and seawater-reared Atlantic salmon. J. Fish Dis..

[2] Gunnarsson G (2017). Temporal changes in infections with some pathogens associated with gill disease in farmed Atlantic salmon (*Salmo salar* L.). Aquaculture.

[3] Mitchell SO, Rodger HD (2011). A review of infectious gill disease in marine salmonid fish. J. Fish Dis..

[4] Oldham T, Rodger H, Nowak BF (2016). Incidence and distribution of amoebic gill disease (AGD) — An epidemiological review. Aquaculture.

[5] Rodger HD, Henry L, Mitchell SO (2011). Non-infectious gill disorders of marine salmonid fish. Rev. Fish Biol. Fish..

[6] Rodger H (2014). Amoebic gill disease (AGD) in farmed salmon (*Salmo salar*) in Europe. Fish Vet. J..

[7] Shinn AP (2015). Economic costs of protistan and metazoan parasites to global mariculture. Parasitology.

[8] Adams MB, Nowak BF (2003). Amoebic gill disease: sequential pathology in cultured Atlantic salmon, *Salmo salar* L. J. Fish Dis..

[9] Adams MB, Ellard K, Nowak BF (2004). Gross pathology and its relationship with histopathology of amoebic gill disease (AGD) in farmed Atlantic salmon, *Salmo salar* L. J. Fish Dis..

[10] Adams MB, Nowak BF (2004). Sequential pathology after initial freshwater bath treatment for amoebic gill disease in cultured Atlantic salmon, *Salmo salar* L. J. Fish Dis..

[11] Roberts S, Powell M (2008). Freshwater bathing alters the mucous layer of marine Atlantic salmon *Salmo salar* L. J. Fish Biol..

[12] Gomez D, Sunyer JO, Salinas I (2013). The mucosal immune system of fish: the evolution of tolerating commensals while fighting pathogens. Fish Shellfish Immunol..

[13] Linden SK, Sutton P, Karlsson NG, Korolik V, McGuckin MA (2008). Mucins in the mucosal barrier to infection. Mucosal Immunol..

[14] Kufe DW (2009). Mucins in cancer: function, prognosis and therapy. Nat. Rev. Cancer.

[15] Rose MC, Voynow JA (2006). Respiratory tract mucin genes and mucin glycoproteins in health and disease. Physiol. Rev..

[16] Jevtov I, Samuelsson T, Yao G, Amsterdam A, Ribbeck K (2014). Zebrafish as a model to study live mucus physiology. Sci. Rep..

[17] Lang T (2016). Searching the evolutionary origin of epithelial mucus protein components - Mucins and FCGBP. Mol. Biol. Evol..

[18] Pérez-Sánchez J (2013). Mucins as diagnostic and prognostic biomarkers in a fish-parasite model: transcriptional and functional analysis. PLoS One.

[19] Marel Mvander (2012). Molecular cloning and expression of two β-defensin and two mucin genes in common carp (*Cyprinus carpio* L.) and their up-regulation after β-glucan feeding. Fish Shellfish Immunol..

[20] Malachowicz M, Wenne R, Burzynski A (2017). De novo assembly of the sea trout (*Salmo trutta* m. *trutta*) skin transcriptome to identify putative genes involved in the immune response and epidermal mucus secretion. PLoS One.

[21] Sveen LR, Grammes FT, Ytteborg E, Takle H, Jorgensen SM (2017). Genome-wide analysis of Atlantic salmon (*Salmo salar*) mucin genes and their role as biomarkers. PLoS One.

[22] Micallef G (2012). Exploring the transcriptome of Atlantic salmon (*Salmo salar*) skin, a major defense organ. Mar. Biotechnol. (NY)..

[23] Benedicenti O, Collins C, Wang T, McCarthy U, Secombes CJ (2015). Which Th pathway is involved during late stage amoebic gill disease?. Fish Shellfish Immunol..

[24] Morrison RN (2007). Molecular cloning and expression analysis of tumour necrosis factor-alpha in amoebic gill disease (AGD)-affected Atlantic salmon (*Salmo salar* L.). Fish Shellfish Immunol..

[25] Morrison RN, Young ND, Nowak BF (2012). Description of an Atlantic salmon (*Salmo salar* L.) type II interleukin-1 receptor cDNA and analysis of interleukin-1 receptor expression in amoebic gill disease-affected fish. Fish Shellfish Immunol..

[26] Pennacchi Y, Leef MJ, Crosbie PBB, Nowak BF, Bridle AR (2014). Evidence of immune and inflammatory processes in the gills of AGD-affected Atlantic salmon, *Salmo salar* L. Fish Shellfish Immunol..

[27] Pennacchi Y, Adams MB, Nowak BF, Bridle AR (2016). Immune gene expression in the gills of Atlantic salmon (*Salmo salar* L.) following experimental reinfection with *Neoparamoeba perurans*. Aquaculture.

[28] Morrison RN (2006). Transcriptome profiling the gills of amoebic gill disease (AGD)-affected Atlantic salmon (*Salmo salar* L.): a role for tumor suppressor p53 in AGD pathogenesis?. Physiol. Genomics.

[29] Wynne JW (2008). Transcriptome analyses of amoebic gill disease-affected Atlantic salmon (*Salmo salar*) tissues reveal localized host gene suppression. Mar. Biotechnol..

[30] Young ND, Cooper GA, Nowak BF, Koop BF, Morrison RN (2008). Coordinated down-regulation of the antigen processing machinery in the gills of amoebic gill disease-affected Atlantic salmon (*Salmo salar* L.). Mol. Immunol..

[31] Marcos-López M (2017). A proteomic approach to assess the host response in gills of farmed Atlantic salmon *Salmo salar* L. affected by amoebic gill disease. Aquaculture.

[32] Yu H, Li Q, Kolosov VP, Perelman JM, Zhou X (2010). Interleukin-13 induces mucin 5AC production involving STAT6/SPDEF in human airway epithelial cells. Cell Commun. Adhes..

[33] Zeng X (2012). Regulation of Interleukin-4 and Interleukin-13 on MUC5B secretion in goblet cells. Chinese Arch. Otolaryngol. - Head Neck Surg..

[34] Burgel P-R, Nadel JA (2004). Roles of epidermal growth factor receptor activation in epithelial cell repair and mucin production in airway epithelium. Thorax.

[35] Takeyama K (1999). Epidermal growth factor system regulates mucin production in airways. Proc. Natl. Acad. Sci. USA.

[36] Cargnello M, Roux PP (2011). Activation and function of the MAPKs and their substrates, the MAPK-activated protein kinases. Microbiol. Mol. Biol. Rev..

[37] McConnell BB, Yang VW (2010). Mammalian Krüppel-like factors in health and diseases. Physiol. Rev..

[38] Lakshmanan I (2015). Mucins in lung cancer: diagnostic, prognostic, and therapeutic implications. J. Thorac. Oncol..

[39] Thornton DJ, Sheehan JK (2004). From mucins to mucus: toward a more coherent understanding of this essential barrier. Proc. Am. Thorac. Soc..

[40] Livraghi-Butrico A (2017). Contribution of mucus concentration and secreted mucins Muc5ac and Muc5b to the pathogenesis of muco-obstructive lung disease. Mucosal Immunol..

[41] Lang T, Hansson GC, Samuelsson T (2007). Gel-forming mucins appeared early in metazoan evolution. Proc. Natl. Acad. Sci. USA.

[42] Peatman E (2013). Basal polarization of the mucosal compartment in *Flavobacterium columnare* susceptible and resistant channel catfish (*Ictalurus punctatus*). Mol. Immunol..

[43] Guardiola FA, Cuesta A, Abellan E, Meseguer J, Esteban MA (2014). Comparative analysis of the humoral immunity of skin mucus from several marine teleost fish. Fish Shellfish Immunol..

[44] Roberts SD, Powell MD (2005). The viscosity and glycoprotein biochemistry of salmonid mucus varies with species, salinity and the presence of amoebic gill disease. J. Comp. Physiol. B..

[45] Roberts SD, Powell MD (2003). Comparative ionic flux and gill mucous cell histochemistry: effects of salinity and disease status in Atlantic salmon (*Salmo salar* L.). Comp. Biochem. Physiol. A. Mol. Integr. Physiol..

[46] Kondo M, Tamaoki J, Takeyama K, Nakata J, Nagai A (2002). Interleukin-13 induces goblet cell differentiation in primary cell culture from Guinea pig tracheal epithelium. Am. J. Respir. Cell Mol. Biol..

[47] Takeyama K, Tamaoki J, Kondo M, Isono K, Nagai A (2008). Role of epidermal growth factor receptor in maintaining airway goblet cell hyperplasia in rats sensitized to allergen. Clin. Exp. Allergy.

[48] Deshmukh HS (2005). Metalloproteinases mediate mucin 5AC expression by epidermal growth factor receptor activation. Am. J. Respir. Crit. Care Med..

[49] Booth BW, Sandifer T, Martin EL, Martin LD (2007). IL-13-induced proliferation of airway epithelial cells: mediation by intracellular growth factor mobilization and ADAM17. Respir. Res..

[50] Shim JJ (2001). IL-13 induces mucin production by stimulating epidermal growth factor receptors and by activating neutrophils. Am. J. Physiol. Lung Cell. Mol. Physiol..

[51] Bridle AR, Morrison RN, Nowak BF (2006). The expression of immune-regulatory genes in rainbow trout, *Oncorhynchus mykiss*, during amoebic gill disease (AGD). Fish Shellfish Immunol..

[52] Heldin C-H, Landstrom M, Moustakas A (2009). Mechanism of TGF-β signaling to growth arrest, apoptosis, and epithelial-mesenchymal transition. Curr. Opin. Cell Biol..

[53] Laster SM, Wood JG, Gooding LR (1988). Tumor necrosis factor can induce both apoptic and necrotic forms of cell lysis. J. Immunol..

[54] Liu M, Yokomizo T (2015). The role of leukotrienes in allergic diseases. Allergol. Int..

[55] Peters-Golden M, Canetti C, Mancuso P, Coffey MJ (2005). Leukotrienes: underappreciated mediators of innate immune responses. J. Immunol..

[56] Secatto A (2012). 5-Lipoxygenase deficiency impairs innate and adaptive immune responses during fungal infection. PLoS One.

[57] Spanbroek R (2001). IL-4 determines eicosanoid formation in dendritic cells by down-regulation of 5-lipoxygenase and up-regulation of 15-lipoxygenase 1 expression. Proc. Natl. Acad. Sci. USA.

[58] Bai Q (2015). Decreased expression of mucin 18 is associated with unfavorable postoperative prognosis in patients with clear cell renal cell carcinoma. Int. J. Clin. Exp. Pathol..

[59] Lin J-C (2014). Significance of expression of human METCAM/MUC18 in nasopharyngeal carcinomas and metastatic lesions. Asian Pac. J. Cancer Prev..

[60] Wu GJ (2001). Expression of a human cell adhesion molecule, MUC18, in prostate cancer cell lines and tissues. Prostate.

[61] Wu G-J, Zeng G (2016). METCAM/MUC18 is a novel tumor and metastasis suppressor for the human ovarian cancer SKOV3 cells. BMC Cancer.

[62] Berman R (2016). MUC18 regulates lung Rhinovirus infection and inflammation. PLoS One.

[63] Schulz C (2003). Upregulation of MCAM in primary bronchial epithelial cells from patients with COPD. Eur. Respir. J..

[64] Simon GC (2011). Up-regulation of MUC18 in airway epithelial cells by IL-13: implications in bacterial adherence. Am. J. Respir. Cell Mol. Biol..

[65] Wang T, Yan J, Xu W, Ai Q, Mai K (2016). Characterization of Cyclooxygenase-2 and its induction pathways in response to high lipid diet-induced inflammation in *Larmichthys crocea*. Sci. Rep..

[66] Kim EK, Choi E-J (2010). Pathological roles of MAPK signaling pathways in human diseases. Biochim. Biophys. Acta.

[67] Morrison DK (2012). MAP kinase pathways. Cold Spring Harb. Perspect. Biol..

[68] Long W (2012). ERK3 signals through SRC-3 coactivator to promote human lung cancer cell invasion. J. Clin. Invest..

[69] Jolodar A, Seghatoleslami S, Seyfiabad Shapouri M, Mesbah M (2011). Identification of a cDNA sequence coding for Krüppel-like factor 2b (Klf2b) from the skin mucosa of common carp (*Cyprinus carpio*). Iran. J. Vet. Res..

[70] Pearson R, Fleetwood J, Eaton S, Crossley M, Bao S (2008). Kruppel-like transcription factors: a functional family. Int. J. Biochem. Cell Biol..

[71] Hu W (2009). Putative tumor-suppressive function of Krüppel-like factor 4 in primary lung carcinoma. Clin. Cancer Res..

[72] Taniguchi H (2012). Silencing of Kruppel-like factor 2 by the histone methyltransferase EZH2 in human cancer. Oncogene.

[73] Yin L (2015). Downregulation of Kruppel-like factor 2 is associated with poor prognosis for nonsmall-cell lung cancer. Tumour Biol..

[74] Yu T (2016). KLF4 regulates adult lung tumor-initiating cells and represses K-Ras-mediated lung cancer. Cell Death Differ..

[75] Xiong Y (2016). Transcription factor KLF2 in dendritic cells downregulates Th2 programming via the HIF-1alpha/Jagged2/Notch axis. MBio.

[76] Das H (2006). Krüppel-like factor 2 (KLF2) regulates proinflammatory activation of monocytes. Proc. Natl. Acad. Sci. USA.

[77] Lu X-J, Chen Q, Chen J, Chen J (2017). Molecular identification and functional analysis of KLF2 in *Plecoglossus altivelis* (ayu): It’s regulatory role in monocyte/macrophage activation. Fish Shellfish Immunol..

[78] Katz JP (2002). The zinc-finger transcription factor Klf4 is required for terminal differentiation of goblet cells in the colon. Development.

[79] Li I-C (2011). Zebrafish Krüppel-like factor 4a represses intestinal cell proliferation and promotes differentiation of intestinal cell lineages. PLoS One.

[80] Loo GH, Sutton DL, Schuller KA (2012). Cloning and functional characterisation of a peroxiredoxin 1 (NKEF A) cDNA from Atlantic salmon (*Salmo salar*) and its expression in fish infected with *Neoparamoeba perurans*. Fish Shellfish Immunol..

[81] Rangasamy T (2005). Disruption of Nrf2 enhances susceptibility to severe airway inflammation and asthma in mice. J. Exp. Med..

[82] Sussan TE (2015). Nrf2 reduces allergic asthma in mice through enhanced airway epithelial cytoprotective function. Am. J. Physiol. Lung Cell. Mol. Physiol..

[83] Zhu L (2008). Identification of Nrf2-dependent airway epithelial adaptive response to proinflammatory oxidant-hypochlorous acid challenge by transcription profiling. Am. J. Physiol. Lung Cell. Mol. Physiol..

[84] Knight PA, Brown JK, Pemberton AD (2008). Innate immune response mechanisms in the intestinal epithelium: potential roles for mast cells and goblet cells in the expulsion of adult *Trichinella spiralis*. Parasitology.

[85] Yamauchi J (2006). Altered expression of goblet cell- and mucin glycosylation-related genes in the intestinal epithelium during infection with the nematode *Nippostrongylus brasiliensis* in rat. APMIS.

[86] Benedicenti O (2017). Characterisation of arginase paralogues in salmonids and their modulation by immune stimulation/infection. Fish Shellfish Immunol..

[87] Diaz A, Allen JE (2007). Mapping immune response profiles: the emerging scenario from helminth immunology. Eur. J. Immunol..

[88] Hallett MA, Venmar KT, Fingleton B (2012). Cytokine stimulation of epithelial cancer cells: the similar and divergent functions of IL4 and IL13. Cancer research.

[89] Kumagai N, Fujitsu Y, Fukuda K, Seki K, Nishida T (2017). Mitogenic and anti-apoptotic effects of IL-4 and IL-13 on human conjunctival fibroblasts. J. Allergy Clin. Immunol..

[90] Taniguchi K (2011). Epigen is induced during the interleukin-13-stimulated cell proliferation in murine primary airway epithelial cells. Exp. Lung Res..

[91] Downes J (2015). A longitudinal study of amoebic gill disease on a marine Atlantic salmon farm utilizing a real-time PCR assay for the detection of *Neoparamoeba perurans*. Aquac. Environ. Interact..

[92] Taylor RS, Muller WJ, Cook MT, Kube PD, Elliott NG (2009). Gill observations in Atlantic salmon (*Salmo salar*, L.) during repeated amoebic gill disease (AGD) field exposure and survival challenge. Aquaculture.

[93] Marcos-López M (2017). Local and systemic humoral immune response in farmed Atlantic salmon (*Salmo salar* L.) under a natural amoebic gill disease outbreak. Fish Shellfish Immunol..

[94] Tamura K, Stecher G, Peterson D, Filipski A, Kumar S (2013). MEGA6: Molecular evolutionary genetics analysis version 6.0. Mol. Biol. Evol..

[95] Livak KJ, Schmittgen TD (2001). Analysis of relative gene expression data using real-time quantitative PCR and the 2(-Delta Delta C(T)) Method. Methods.

