# Supplementary material for: Gene expression analysis of Atlantic salmon gills reveals mucin 5 and interleukin 4/13 as key molecules during amoebic gill disease
Source: Sci Rep. 2018 Sep 12;8:13689. doi: 10.1038/s41598-018-32019-8 (PMC6135806; doi:10.1038/s41598-018-32019-8)
Supplement: Supplementary file 1 — Supplementary Tables [file 41598_2018_32019_MOESM1_ESM.pdf]

Gene expression analysis of Atlantic salmon gills reveals mucin 5 and interleukin 4/13 as key molecules during amoebic gill disease

Mar Marcos-López[1,2,*], Josep A. Calduch-Giner[3], Luca Mirimin[1], Eugene MacCarthy[1], Hamish D. Rodger[2], Ian O'Connor[1], Ariadna Sitjà-Bobadilla[4], Jaume Pérez-Sánchez[3], M. Carla Piazzon[4,*]

[1]*Marine and Freshwater Research Centre, Galway-Mayo Institute of Technology, Dublin Road, Galway, Co. Galway, H91 T8NW, Ireland.*

[2]*FishVet Group Ireland, Unit 7b Oranmore Business Park, Oranmore, Co. Galway, H91 XP3F, Ireland.*

[3]*Nutrigenomics and Fish Growth Endocrinology Group, Instituto de Acuicultura Torre de la Sal (IATS-CSIC), Castellón, 12595, Spain.*

[4]*Fish Pathology Group, Instituto de Acuicultura Torre de la Sal (IATS-CSIC), Castellón, 12595, Spain.*

[*]*Corresponding authors: Mar Marcos-López, E-mail address: mar.marcos.lopez@gmail.com // M. Carla Piazzon, E-mail address: carla.piazzon@csic.es*

Supplementary Table S1: Contributions (%) of the different genes studied to the variability of dimensions (principal component, PC) 1 and 2 of the PCA analyses in Fig. 5 (A), Fig. 6A (B) and Fig. 6B (C).

**A**	**Contributions (%)**	
**Gene**	**PC1**	**PC2**
muc18	0,03	0,00
muc5	98,39	0,51
muc2	0,02	0,00
tnfα3	0,03	0,02
il4/13a	0,09	81,88
il4/13b2	1,30	12,28
tgfβ1b	0,01	0,07
p38b	0,00	0,24
mapk6 (erk3)	0,00	0,03
mapk8 (jnk1)	0,00	0,17
nrf2	0,01	0,01
hsp70	0,00	0,78
phb	0,02	0,86
ppia	0,01	0,45
pcna	0,02	1,57
egfr	0,01	0,22
p53	0,00	0,07
klf4	0,00	0,14
klf11	0,01	0,04
klf2	0,01	0,16
lox5	0,02	0,50

**B**	**Contributions (%)**	
**Gene**	**PC1**	**PC2**
muc18	0,20	0,00
muc5	48,74	36,27
muc2	31,83	57,28
tnfα3	0,72	0,93
il4/13a	4,19	1,17
il4/13b2	12,66	0,75
tgfβ1b	0,23	0,33
p38b	0,14	0,13
mapk6 (erk3)	0,01	0,00
mapk8 (jnk1)	0,15	0,02
nrf2	0,29	0,02
hsp70	0,41	1,47
phb	0,00	0,30
ppia	0,01	0,03
pcna	0,00	0,22
egfr	0,00	0,00
p53	0,04	0,03
klf4	0,24	0,22
klf11	0,11	0,45
klf2	0,00	0,01
lox5	0,04	0,37

**C**	**Contributions (%)**	
**Gene**	**PC1**	**PC2**
muc18	0,02	0,01
muc5	48,59	39,50
muc2	33,85	57,48
tnfα3	0,31	0,21
il4/13a	4,98	1,16
il4/13b2	10,69	0,34
tgfβ1b	0,07	0,03
p38b	0,02	0,09
mapk6 (erk3)	0,08	0,00
mapk8 (jnk1)	0,01	0,01
nrf2	0,08	0,03
hsp70	1,08	0,22
phb	0,04	0,17
ppia	0,05	0,02
pcna	0,03	0,23
egfr	0,03	0,00
p53	0,00	0,00
klf4	0,05	0,00
klf11	0,00	0,13
klf2	0,02	0,28
lox5	0,02	0,07

Supplementary Table S2: Relative gene expression values of fish from the *in vivo* challenge. β-actin was used as a housekeeping gene and all values were referenced to the expression level of *egfr* from non-infected fish at 2 days post-infection (dpi) with an arbitrarily assigned value of 1. Empty cells are excluded values due to inconsistent results or melting curves.

Gene	2 dpi non-infected (negative)						Mean	2 dpi infected (positive)						Mean
muc18	3.16	2.66	2.45	1.80	2.33	2.28	2.45	3.25	3.84	1.93	2.99	2.35	2.57	2.82
muc5	0.04	0.02	0.13	0.08	0.06	0.03	0.06	0.03	0.07	0.11	0.10	0.04	0.12	0.08
muc2	4.37E-04	2.23E-03	2.02E-03	1.31E-03	1.93E-04	1.52E-04	1.06E-03	9.63E-04	1.72E-03	3.01E-04	3.55E-04	1.53E-04	2.82E-04	6.30E-04
tnfα3	0.11	0.15	0.10		0.14	0.12	0.12	0.12	0.18	0.14	0.11	0.12	0.10	0.13
il4/13a	0.58	0.50	0.76	0.50	0.74	0.35	0.57	0.75	1.14	0.65	1.09	0.97	1.64	1.04
il4/13b2	0.02	0.02	0.02	0.02	0.02	0.02	0.02	0.02	0.03	0.03	0.04	0.03	0.04	0.03
tgfß1b	0.45	0.51	0.77	0.34	0.52	0.53	0.52	0.80	0.88	0.62	0.56	0.62	0.48	0.66
p38b	7.89	7.89	8.57	4.56	6.28	5.98	6.86	8.22	8.57	6.59	8.06	6.50	6.41	7.39
mapk6 (erk3)	1.61	2.07	1.61	0.95	1.58	1.64	1.58	1.67	1.83	1.42	1.69	1.59	1.45	1.61
mapk8 (jnk1)	1.18	1.49	1.14	1.13	1.34	1.13	1.23	1.53	1.65	1.01	1.47	1.05	1.27	1.33
nrf2	6.02	10.06	5.86	4.20	6.50	6.41	6.51	6.82	7.84	5.24	7.41	6.11	5.50	6.49
hsp70	0.39	0.63	0.25	0.66	0.44	0.28	0.44	0.50	0.41	0.55	0.40	0.23	0.40	0.42
phb	5.66	9.06	7.31	4.23	6.63	7.62	6.75	8.00	8.46	5.31	7.21	6.28	6.59	6.97
ppia	23.92	33.36	23.10	15.45	50.21	28.25	29.05	31.56	33.36	24.25	26.35	18.90	22.63	26.17
pcna	3.23	5.54	3.97	2.25	3.46	6.02	4.08	5.94	5.13	4.14	3.78	3.78	3.76	4.42
egfr	1.09	1.74	1.22	0.64	0.64	1.07	1.07	0.99	1.16	0.75	1.13	0.84	0.81	0.94
p53	2.71	3.92	3.18	2.41	2.97	3.51	3.12	3.53	3.63	3.32	3.48	2.87	3.16	3.33
klf4	0.54	0.80	0.36	0.40	0.57	0.54	0.53	0.41	0.57	0.43	0.76	0.58	0.59	0.56
klf11	1.16	0.93	0.72	0.62	0.52	1.23	0.87	1.12	1.60	0.68	1.47	1.01	0.95	1.14
klf2	3.20	4.35	2.97	2.69	2.69	3.29	3.20	2.97	4.23	3.20	5.21	3.51	4.08	3.87
lox5	2.33	1.69	1.41	1.40	1.78	1.31	1.65	2.31	2.68	2.81	2.53	1.64	1.80	2.29

Gene	7 dpi non-infected (negative)						Mean	7 dpi infected (positive)						Mean
muc18	1.77	2.48	2.38	2.39	2.33	2.28	2.27	2.14	1.96	2.30	2.23	2.35	2.57	2.26
muc5	0.06	0.22	0.13	0.01	0.06	0.03	0.09	0.07	0.26	0.05	0.26	0.04	0.12	0.13
muc2	2.66E-02	7.88E-04	7.40E-04	5.31E-04	1.93E-04	1.52E-04	4.84E-03	9.97E-04	4.34E-04	5.13E-04	6.81E-04	1.53E-04	2.82E-04	5.10E-04
tnfα3	0.11	0.15	0.13	0.06	0.14	0.12	0.12	0.09	0.08	0.10	0.10	0.12	0.10	0.10
il4/13a	0.16	0.22	0.69	0.13	0.74	0.35	0.38		1.57	0.78	1.74	0.97	1.64	1.34
il4/13b2	0.03	0.02	0.02	0.01	0.02	0.02	0.02		0.06	0.02	0.03	0.03	0.04	0.04
tgfß1b	0.18	0.66	0.54	0.56	0.52	0.53	0.50	0.44	0.55	0.49	0.49	0.62	0.48	0.51
p38b	4.11	5.39	5.31	5.86	6.28	5.98	5.49	5.50	4.86	5.62	6.36	6.50	6.41	5.87
mapk6 (erk3)	1.46	1.78	1.43	1.17	1.58	1.64	1.51	2.08	1.19	1.24	1.93	1.59	1.45	1.58
mapk8 (jnk1)	1.09	1.28	1.05	1.07	1.34	1.13	1.16	1.21	1.05	1.10	1.13	1.05	1.27	1.14
nrf2	4.76	5.86	5.13	5.17	6.50	6.41	5.64	6.50	3.97	5.86	5.50	6.11	5.50	5.57
hsp70	0.39		0.40	0.29	0.44	0.28	0.36	0.57	0.31	0.31	0.57	0.23	0.40	0.40
phb	2.97	6.50	5.78	5.39	6.63	7.62	5.81	9.58	6.59	5.54	9.78	6.28	6.59	7.39
ppia	21.41	22.78	21.41	17.15		28.25	22.20	29.45	22.16	17.15	25.81	18.90	22.63	22.68
pcna	2.25	4.35	3.94	2.58	3.46		3.32	4.76	5.10	2.95	6.23	3.78	3.76	4.43
egfr	0.95	1.24	0.68	0.94	0.64	1.07	0.92	1.49	0.55	0.78	0.95	0.84	0.81	0.90
p53	2.66	3.01	3.20	2.83	2.97	3.51	3.03	3.43	2.64	3.16	4.26	2.87	3.16	3.25
klf4	0.53	0.51	0.57	0.58	0.57	0.54	0.55		0.50	0.31	0.51	0.58	0.59	0.50
klf11	1.04	0.81	0.42	0.61	0.52	1.23	0.77	0.52	0.49	0.90	0.93	1.01	0.95	0.80
klf2	4.00	4.50	3.16		2.69	3.29	3.53	3.23	1.80	2.69	3.94	3.51	4.08	3.21
lox5	1.87	2.04	1.92	1.80	1.78	1.31	1.79	0.97	1.75	1.87	2.06	1.64	1.80	1.68

Supplementary Table S2: Continued.

Gene	14 dpi non-infected (negative)						Mean	14 dpi infected (positive)						Mean
muc18	2.89	2.25	1.65	2.38	2.43	1.88	2.25	2.38	2.79	2.85	2.08	1.92	1.87	2.31
muc5	0.02	0.04	0.08	0.03	0.01	0.01	0.03	0.29	0.07	0.19	0.17	0.35	0.47	0.25
muc2	5.42E-04	7.45E-04	8.14E-03	2.23E-04	4.54E-02	1.49E-02	1.17E-02	3.29E-04	1.39E-03	8.44E-04	2.20E-04	6.96E-05	5.85E-05	4.85E-04
tnfα3	0.14	0.16	0.09	0.25	0.13		0.15	0.08	0.13	0.09	0.09	0.09	0.06	0.09
il4/13a	1.17	0.47	1.34	0.48	0.31	1.09	0.81	2.20		1.83	1.61	1.88	1.82	1.87
il4/13b2	0.02	0.02	0.05	0.02	0.04	0.05	0.03	0.06	0.02	0.06	0.06	0.04	0.05	0.05
tgfß1b	0.62	0.44	0.32	0.70	0.57	0.41	0.51	0.55	0.66	0.55	0.52	0.44	0.50	0.54
p38b	7.11	5.74	9.19	6.92	4.89	4.53	6.40	5.43	7.73	6.23	6.28	4.92	4.92	5.92
mapk6 (erk3)	1.32	0.98	1.82	1.21	2.19	1.74	1.54	1.75	1.80	1.74	2.23	1.62	1.87	1.84
mapk8 (jnk1)	1.46	1.10	1.27	1.33	1.37	1.49	1.34	1.05	1.36	1.28	1.11	0.93	1.06	1.13
nrf2	6.59	4.59	5.94	5.86	8.22	7.57	6.46	4.76	7.46	6.28	5.50	5.06	4.17	5.54
hsp70	0.51	0.26	0.38	0.47	0.47	0.41	0.42	0.36	0.38	0.45	0.46	0.52	0.46	0.44
phb	5.06	4.06	6.54	6.15	3.89	3.92	4.94	8.75	8.82	7.01	8.17	6.19	8.06	7.83
ppia	22.78	18.64	26.17	18.38	24.08	26.35	22.74	29.86	28.05	26.72	23.92	22.47	25.81	26.14
pcna	3.89	2.31	3.92	3.20	2.00	2.51	2.97	5.24	4.06	4.38	4.96	3.94	5.82	4.73
egfr	0.81	0.57	1.03	0.81	0.91	0.87	0.83	1.01	1.16	0.89	1.02	1.00	0.78	0.98
p53	3.05	2.51	3.51	3.66	2.93	3.14	3.13	3.27	3.51	3.48	3.32	3.05	3.48	3.35
klf4	0.53	0.38	0.44		0.40	0.44	0.44	0.31	0.41	0.38	0.32	0.35	0.37	0.36
klf11	0.80	0.93	0.94	0.59	0.86	0.59	0.79	0.67	0.95		0.95	0.90	0.49	0.79
klf2	4.50	3.27	2.89	4.38	3.84	4.44	3.89	2.87	3.73	4.06	4.06	3.68	2.97	3.56
lox5	2.66	1.99	2.11	2.81	2.39	2.57	2.42	2.48	2.38	2.81	2.81	2.53	1.75	2.46

Gene	21 dpi non-infected (negative)						Mean	21 dpi infected (positive)						Mean
muc18	2.38	2.22	2.48	2.95	2.39	1.72	2.36		1.39	1.47	1.42	1.12	1.20	1.32
muc5	0.03	0.02	0.03	0.05	0.02	0.02	0.03	0.81	1.06	0.76	0.57	0.84	0.68	0.79
muc2	7.20E-04	1.51E-03	2.90E-04	2.49E-04	3.78E-04	1.59E-04	5.51E-04	6.81E-04	0.00E+00	0.00E+00	6.18E-04	3.81E-05	0.00E+00	4.46E-04
tnfα3	0.09	0.08	0.14	0.16	0.07	0.13	0.11	0.07	0.05	0.06	0.04	0.05	0.05	0.05
il4/13a	0.42	0.14	0.19	0.69	0.28	0.53	0.38	1.48	1.64	1.36	1.14	0.95	0.78	1.22
il4/13b2	0.02	0.02	0.02	0.02	0.01	0.02	0.02	0.12	0.12	0.07	0.06	0.06		0.09
tgfß1b	0.46	0.56	0.55	0.59	0.38	0.57	0.52	0.48	0.48	0.42	0.36	0.34	0.37	0.41
p38b	4.44	6.73	6.68	6.92	4.69	4.76	5.70	6.68	6.73	5.21	5.62	5.13	5.03	5.73
mapk6 (erk3)	1.09	1.49	1.60	1.66	0.99	1.13	1.33		1.67	1.45	1.84	1.75	1.64	1.67
mapk8 (jnk1)	0.84	1.38	1.21	1.52	0.93	0.98	1.14	1.13	1.12	0.91	1.14	0.85	0.90	1.01
nrf2	4.72	6.11	6.73	7.52	4.76	4.41	5.71	4.63	3.81	3.73	4.44	3.97	3.39	3.99
hsp70	0.39	0.27	0.41	0.41	0.28	0.38	0.36	0.45	0.30	0.42	0.31	0.32	0.22	0.34
phb	4.23	6.54	6.82	6.50	4.29	4.38	5.46	7.67	11.71	8.75	7.31	7.62	6.11	8.20
ppia	17.15	28.44	24.59	25.11	15.56	19.03	21.65	30.48	31.12	21.26	26.54	23.92	22.78	26.02
pcna	2.83	3.34	3.43	3.61	2.66	3.86	3.29	5.62	7.11	5.17	4.76	4.59	3.41	5.11
egfr	0.50		0.66	0.78	0.50	0.49	0.59	0.77	0.76	0.65	0.97	0.82	1.20	0.86
p53	2.28	2.87	3.14	3.78	1.88	2.73	2.78	3.51	3.46	3.01	3.81	2.93	3.27	3.33
klf4	0.21	0.41	0.49		0.34	0.36	0.36	0.40	0.18	0.26	0.48	0.21	0.36	0.32
klf11	0.71	1.05	0.55	0.69	0.29	0.42	0.62	0.57	0.55	0.65	0.35	0.60	0.32	0.51
klf2	2.30	3.36	2.64	3.84	2.19	2.46	2.80	1.85	1.62	2.19	2.30	2.14		2.02
lox5	2.60	2.38	2.07	2.81	1.96	1.71	2.25	1.74	1.73	1.71	2.07	1.67	1.65	1.76

Supplementary Table S3: Relative gene expression values of fish from the field sampling. β-actin was used as a housekeeping gene and all values were referenced to the expression level of *egfr* in GS 0 with an arbitrarily assigned value of 1. Empty cells are excluded values due to inconsistent results or melting curves.

Gene	GS0					Mean
muc18	2.83	4.63	3.56	3.73	2.58	3.47
muc5	0.21	0.20	0.08	0.14	0.06	0.14
muc2	4.34E-04	1.47E-03	1.53E-04	4.59E-04	1.28E-03	7.59E-04
tnfα3	0.10	0.16	0.09	0.15	0.13	0.13
il4/13a	0.15	0.34	0.28	0.23	0.23	0.25
il4/13b2	0.02	0.03	0.02	0.04	0.02	0.02
tgfß1b	0.47	0.66	0.48	0.63	0.55	0.56
p38b	6.11	10.85	8.46	10.78	4.72	8.18
mapk6 (erk3)	1.30	2.22	2.03	2.87	1.82	2.05
mapk8 (jnk1)	1.16	2.48	1.69	2.43	1.78	1.91
nrf2	6.63	8.51	8.51	8.82	7.16	7.93
hsp70	0.35	0.62	0.36	0.45	0.39	0.43
phb	5.50	11.31	10.27	9.51	6.63	8.65
ppia	23.26	36.76	25.81	29.65	27.28	28.55
pcna	3.94	7.84	5.58	5.43	5.94	5.74
egfr	0.60	1.28	1.07	1.34	0.91	1.04
p53	2.83	5.24	4.47	5.28	3.89	4.34
klf4	0.51	1.04	1.16	1.20	0.66	0.91
klf11	0.74	1.51	1.46	1.30	0.90	1.18
klf2	3.97	7.36	7.67	9.65	5.46	6.82
lox5	1.30	1.74	1.34	2.00	1.38	1.55

Gene	GS1 Lesion					Mean	GS1 Non-lesion					Mean
muc18	3.32	3.03	3.05	3.48	2.87	3.15	2.85	2.45	2.53	3.14	2.97	2.79
muc5	0.29	0.21	0.74	0.17	0.57	0.39	0.11	0.10	0.08	0.02	0.01	0.06
muc2	1.36E-03	1.11E-03	1.53E-03	2.12E-03	1.19E-03	1.46E-03	7.20E-04	2.08E-03	1.89E-03	2.46E-03	1.84E-03	1.80E-03
tnfα3	0.11	0.22	0.14	0.24	0.16	0.17	0.13	0.14	0.16	0.26	0.15	0.17
il4/13a	0.35	0.43	0.29	0.38	0.50	0.39	0.31	0.33	0.21	0.27	0.24	0.27
il4/13b2	0.04	0.05	0.04		0.05	0.04	0.03	0.07	0.06	0.05	0.04	0.05
tgfß1b	0.59	0.73	0.67	0.77	0.78	0.71	0.52	0.56	0.58	0.72	0.61	0.60
p38b	5.54	7.36	7.06	6.87	6.23	6.61	5.86	6.68	6.63	6.82	5.78	6.35
mapk6 (erk3)	2.20	2.16	1.92	3.12	2.17	2.31	2.14	2.53	2.00	2.48	1.64	2.16
mapk8 (jnk1)	1.32	1.60	1.16	1.82	1.60	1.50	1.42	1.62	1.31	1.55	1.21	1.42
nrf2	9.00	8.34	6.63	11.00	8.11	8.62	9.25	9.51	8.17	8.88	8.63	8.89
hsp70	0.43	0.18	0.83	0.53	0.41	0.48	0.50	0.29	0.36	0.46	0.51	0.42
phb	8.06	8.75	7.52	8.88	11.08	8.86	6.28	7.94	6.63	6.19	6.19	6.65
ppia	30.91	35.26	24.93	37.27	40.22	33.72	33.13	34.54	25.46	30.91	26.35	30.08
pcna	5.86	7.01	7.21	5.50	8.63	6.84	5.35	6.02	4.72	5.54	5.98	5.52
egfr	1.04	1.40	0.93	1.44	1.13	1.19	1.23	1.13	0.77	1.27	1.04	1.09
p53	4.32	4.59	4.41	5.46	4.69	4.69	3.94	4.82	4.08	4.56	3.81	4.25
klf4	0.64	0.81	0.70	0.94	0.55	0.73	0.78	0.93	0.80	1.01	0.68	0.84
klf11	0.75	1.29	0.98	0.71	1.01	0.95	0.85	1.23	1.01	0.71	1.09	0.98
klf2	13.55	9.58	8.69	15.35	10.13	11.46	13.74	11.24	10.70	14.22	13.36	12.65
lox5	1.67	2.57	2.41	3.05	2.62	2.46	1.88	2.35	2.45	2.58	2.10	2.27

Supplementary Table S3: Continued.

Gene	GS2 Lesion					Mean	GS2 Non-lesion					Mean
muc18	2.43	3.27	2.33	1.84	2.73	2.52	2.08	3.34	3.05	2.85	3.48	2.96
muc5	0.44	0.99	0.27	0.44	0.90	0.61	0.15	0.11	0.05	0.04	0.04	0.08
muc2	1.29E-03	1.55E-03	1.41E-03	7.49E-03	2.94E-03	2.94E-03		2.21E-03	5.23E-04	2.49E-03	3.26E-03	2.12E-03
tnfα3	0.13	0.18	0.17	0.10	0.17	0.15	0.14	0.21	0.26	0.25	0.10	0.19
il4/13a	0.55	0.87	0.33	0.54	0.43	0.54	0.28	0.39	0.27	0.27	0.19	0.28
il4/13b2	0.08	0.11		0.11	0.07	0.09	0.03	0.07	0.05	0.06	0.04	0.05
tgfß1b	0.63	0.77	0.44	0.48	0.66	0.60	0.42	0.57	0.64	0.63	0.50	0.55
p38b	4.89	5.90	4.23	3.63	7.21	5.17	4.14	5.13	6.54	6.59	5.78	5.64
mapk6 (erk3)	1.84	2.71	1.66	2.00	2.20	2.08	1.56	2.33	1.91	2.10	1.72	1.92
mapk8 (jnk1)	1.12	1.39	0.96	0.93	1.39	1.16	1.13	1.58	1.26	1.49	1.27	1.35
nrf2	6.32	6.54	6.41	4.89	6.54	6.14	6.77	9.92	7.46	8.82	8.00	8.19
hsp70	0.11	0.65	0.46	0.34	0.48	0.41	0.10	0.70	0.40	0.44	0.47	0.42
phb	9.06	8.69	5.74	6.59	7.46	7.51	4.44	5.50	5.98	9.45	7.16	6.51
ppia	34.78	35.02	21.71	27.67	34.06	30.64	28.05	32.22	27.28	31.12	29.24	29.59
pcna	6.28	6.32	4.14	4.41	5.62	5.35	3.29	3.58	4.99	5.66	4.59	4.42
egfr	0.93	1.07	0.81	0.95	1.14	0.98	0.80	1.11	1.06	1.05	1.14	1.03
p53	3.89	3.71	3.29	3.73	4.76	3.88	3.16	3.92	3.84	5.06	3.36	3.87
klf4	0.40	0.55	0.53	0.26	0.53	0.45	0.63	0.77	0.77	0.89	0.64	0.74
klf11	0.53	0.50	0.41	0.44	0.64	0.51	0.47	0.40	0.57	0.56	0.46	0.49
klf2	7.31	7.36	5.43	6.23	8.94	7.05	10.48	10.41	7.11	12.21	8.40	9.72
lox5	2.22	2.30	1.84	1.83	2.20	2.08	2.14	2.16	1.82	2.53	1.72	2.07

Gene	Post-treatment Lesion					Mean	Post-treatment Non-lesion					Mean
muc18	3.03	3.48	3.86	5.78	3.29	3.89	2.48	3.29	1.47	1.27	2.39	2.18
muc5	0.03	0.08	0.06	0.09	0.02	0.06	0.02	0.10	0.07	0.07	0.10	0.07
muc2	7.99E-04	1.75E-03	1.28E-03		2.12E-03	1.49E-03	2.84E-04	6.81E-04	1.73E-05	1.62E-03	2.00E-04	5.60E-04
tnfα3	0.33	0.33	0.33	0.33	0.21	0.31	0.16	0.29	0.12	0.15	0.14	0.17
il4/13a	0.28	0.20	0.23	0.21	0.23	0.23	0.16	0.15	0.12	0.08	0.20	0.14
il4/13b2	0.04	0.04	0.05	0.04	0.06	0.05	0.03	0.04	0.04	0.02	0.05	0.04
tgfß1b	0.80	1.03	1.77	1.06	0.91	1.11	0.44	0.82	0.85	0.67	0.69	0.69
p38b	7.89	8.11	5.58	7.89	4.89	6.87	4.23	6.68	4.50	3.71	5.62	4.95
mapk6 (erk3)	1.66	1.69	2.66	2.79	1.65	2.09	1.12	1.54	1.66	1.78	1.77	1.57
mapk8 (jnk1)	1.40	1.99	1.47	1.78	0.95	1.52	0.82	1.37	0.76	0.70	1.13	0.96
nrf2	8.51	8.94	11.00	11.79	7.57	9.56	5.74	7.36	5.35	4.38	7.57	6.08
hsp70	0.45	0.30	1.74	1.34	1.29	1.02	0.35	0.29	1.33	1.03	1.87	0.97
phb	5.10	6.96	6.96	7.57	3.61	6.04	3.23	5.58	8.06	9.92	6.50	6.66
ppia	29.45	34.54	34.54	35.51	21.86	31.18	20.11	35.26	23.75	29.04	30.06	27.65
pcna	4.69	6.68	6.02	7.16	4.03	5.72	3.39	4.63	5.46	6.15	4.38	4.80
egfr	0.82	1.27	1.10	1.35	0.64	1.04	0.60	0.84	0.76	0.68	0.72	0.72
p53	3.86	4.66	5.98	6.45	4.32	5.05	2.43	3.63	3.56	3.94	3.71	3.45
klf4	0.47	0.39	0.51	0.77	0.44	0.52	0.30	0.44	0.30	0.19	0.55	0.36
klf11	0.40	0.44	0.48	0.53	0.23	0.42	0.22	0.29	0.37	0.30	0.25	0.29
klf2	5.66	7.67	9.06		8.82	7.80	3.71	5.24	6.73	6.54	8.34	6.11
lox5	2.77	3.39	3.36	4.20	2.69	3.28	2.00	2.51	1.30	1.07	1.85	1.75
